# Shifts in microbial community, pathogenicity‐related genes and antibiotic resistance genes during dairy manure piled up

**DOI:** 10.1111/1751-7915.13551

**Published:** 2020-03-23

**Authors:** Xu Zhang, Chenjie Ma, Wen Zhang, Wu Li, Jialin Yu, Di Xue, Xiaolin Wu, Guangcun Deng

**Affiliations:** ^1^ Key Lab of Ministry of Education for Protection and Utilization of Special Biological Resources in Western China Ningxia University Ningxia China; ^2^ School of Life Science Ningxia University Ningxia China

## Abstract

The uncomposted faeces of dairy cow are usually stacked on cow breeding farms, dried under natural conditions and then used as cow bedding material or they may be continuously piled up. However, no information is available to evaluate variations in the human and animal pathogen genes and antibiotic resistance during the accumulation of fresh faeces of dairy cow to manure. Here, we present the metagenomic analysis of fresh faeces and manure from a dairy farm in Ning Xia, showing a unique enrichment of human and animal pathogen genes and antibiotic resistance genes (ARGs) in manure. We found that manure accumulation could significantly increase the diversity and abundance of the pathogenic constituents. Furthermore, pathogens from manure could spread to the plant environment and enphytotic pathogens could affect the yield and quality of crops during the use of manure as a fertilizer. Levels of virulence genes and ARGs increased with the enrichment of microbes and pathogens when faeces accumulated to manure. Accumulated manure was also the transfer station of ARGs to enrich the ARGs in the environment, indicating the ubiquitous presence of environmental antibiotic resistance genes. Our results demonstrate that manure accumulation and usage without effective manure management is an unreasonable approach that could enrich pathogenic microorganisms and ARGs in the environment. The manure metagenome structure allows us to appreciate the overall influence and interaction of animal waste on water, soil and other areas impacted by faecal accumulation and the factors that influence pathogen occurrence in products from dairy cows.

## Graphical abstract

Manure accumulation could significantly increase the diversity and abundance of the microbial and pathogenic constituents in the tested manure. The diversity and abundance of microbial and pathogenic constituents were significantly higher in manure than in fresh faeces. Levels of virulence genes and ARGs increased with the enrichment of microbes and pathogens when faeces accumulated to manure. Manure accumulation and usage without effective manure management is an unreasonable approach that could enrich pathogenic microorganisms and ARGs in the environment.

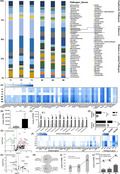

## Introduction

Management of organic waste is an important problem in cow breeding, particularly the faecal waste in a dairy farm. Composting has been recognized as an effective technique for the treatment of organic wastes (Ran *et al.*, 2017). However, composting requires specific processes including temperature, environmental conditions and special additives to remove harmful substances in faeces, such as harmful gases, heavy metals, parasite eggs, pathogens and antibiotic resistance genes (ARGs) (Huang *et al.*, 2017). Unfortunately, with the increase of people's demand for dairy products and the expansion of cow breeding scale, faeces are produced faster than they can be composted. The remaining faeces are usually piled up on cow breeding farms, dried under natural conditions and then used as cow bedding material or continuously piled up without effective management (Blowey *et al.*, 2013). In this process, the harmful ingredients, including virulence genes and ARGs of pathogens in faeces, may endanger the food safety of dairy products by integrating into the human microbiome and make humans drug‐resistant (Hoelzer *et al.*, 2017).

Pathogens and antibiotic residues are the major harmful compositions in manure (faeces accumulated under natural conditions). According to the statistics of the World Health Organization (WHO), there are approximately 1.5 billion diarrhoea patients in the world every year, and 70% of those cases were caused by food contaminated with microorganisms (Akeda, 2015). Delahoy *et al.* (2018) reported that most pathogenic bacteria in food may resulting from the excretion of livestock and poultry faeces; the pathogens can survive in water, soil and other areas of environments for a long time and then affect human health through crops or animal products. At present, more than 150 human or animal pathogens have been identified in faeces (Mcdaniel *et al.*, 2014). Thus, it is necessary to investigate the characterization of pathogenic microorganisms in fresh faeces and manure.

In China, nearly half of the antibiotics consumed (162 000 tons in 2013) are used in animal husbandry, of which substantial amounts could end up in manure (Zhang *et al.*, 2015). The widespread usage of antibiotics in the livestock and poultry industries could lead to diseases and promote the abundances of antibiotic‐resistant bacteria and ARGs increasing in the environment (Hu and Cheng, 2016). High concentrations of antibiotics and ARGs have been found in livestock faeces (Cheng *et al.*, 2014; Karkman *et al.*, 2019). In addition, there is a growing risk of ARGs spreading to pathogens via horizontal gene transfer (HGT), which could make antibiotics vestigial even if the bacteria carrying ARGs die (Wintersdorff *et al.*, 2016), as some ARGs may persist even when the antibiotic pressure is gone (Zhao *et al.*, 2017; Zhang *et al.*, 2018). The hazards of piled up cow manure have received less attention, despite the fact that it is widely used for crop or vegetables production in china. Although dairy cows often have a lower antibiotic usage intensity than other meat‐producing animals (Wang *et al.*, 2017), cow manure was also found to contain diverse ARGs (Noyes *et al.*, 2016; Zhou *et al.*, 2016). The spread of ARGs to pathogens can restrict, the therapeutic potential of antibiotics, thereby posing a potential threat to the health of humans and livestock. However, little information is available with respect to the levels and changes in pathogenic bacteria and ARGs when faeces accumulate to manure in dairy cow farms.

In the present study, we provide insights into the variations in the microbial community, pathogens and ARGs when fresh faeces accumulate to manure. The accumulation of manure in dairy farms without effective manure management would lead to the enrichment of pathogenic bacteria and ARGs, increase the risk of diseases transmitted by faecal contamination, and may threaten the health of dairy cows and the safety of dairy products.

## Results

### Findings from the metagenomic raw data

Metagenomic raw data (mean ± SEM, raw reads, group F: 1.174E + 8±0.957E + 7, group M: 1.783E + 8±0.541E + 8) were filtered to clean data (mean ± SEM, clean reads, group F: 1.171E + 8±0.953E + 7, group M: 1.776E + 8±0.541E + 8) and assembled. We obtained an average of 241.6 ± 37.0 k (mean ± SEM, k‐ kilo) and 308.9 ± 90.1 k (mean ± SEM, k‐ kilo) counts for fresh faeces and manure samples (Table S1). The Bray–Curtis distance of relative abundance (RA) suggests that there are differences and diversity between fresh faeces and manure samples. The length of distribution of cleaned reads is shown in Fig. S1 and Table S1. Taken together, these data then being used in a subsequent analysis for changes in the microbial community, pathogenicity and antibiotic resistance between fresh faeces and manure.

### The diversity composition of microorganisms in faeces

A total of 1 651 686 high‐quality assembled counts were generated, the counts were then aligned against available microbial genomes from the NCBI‐NR database. Taxonomic composition was generated for all samples at the levels of kingdom, phylum, class, order, family, genus and species, as shown in Table S2. The results indicated that Bacteria (including Archaea, Bacteria, Eukaryota and Viruses) was predominant in fresh faeces samples (97.29%) and manure samples (97.49%) at the kingdom level.

**Fig. 1:**
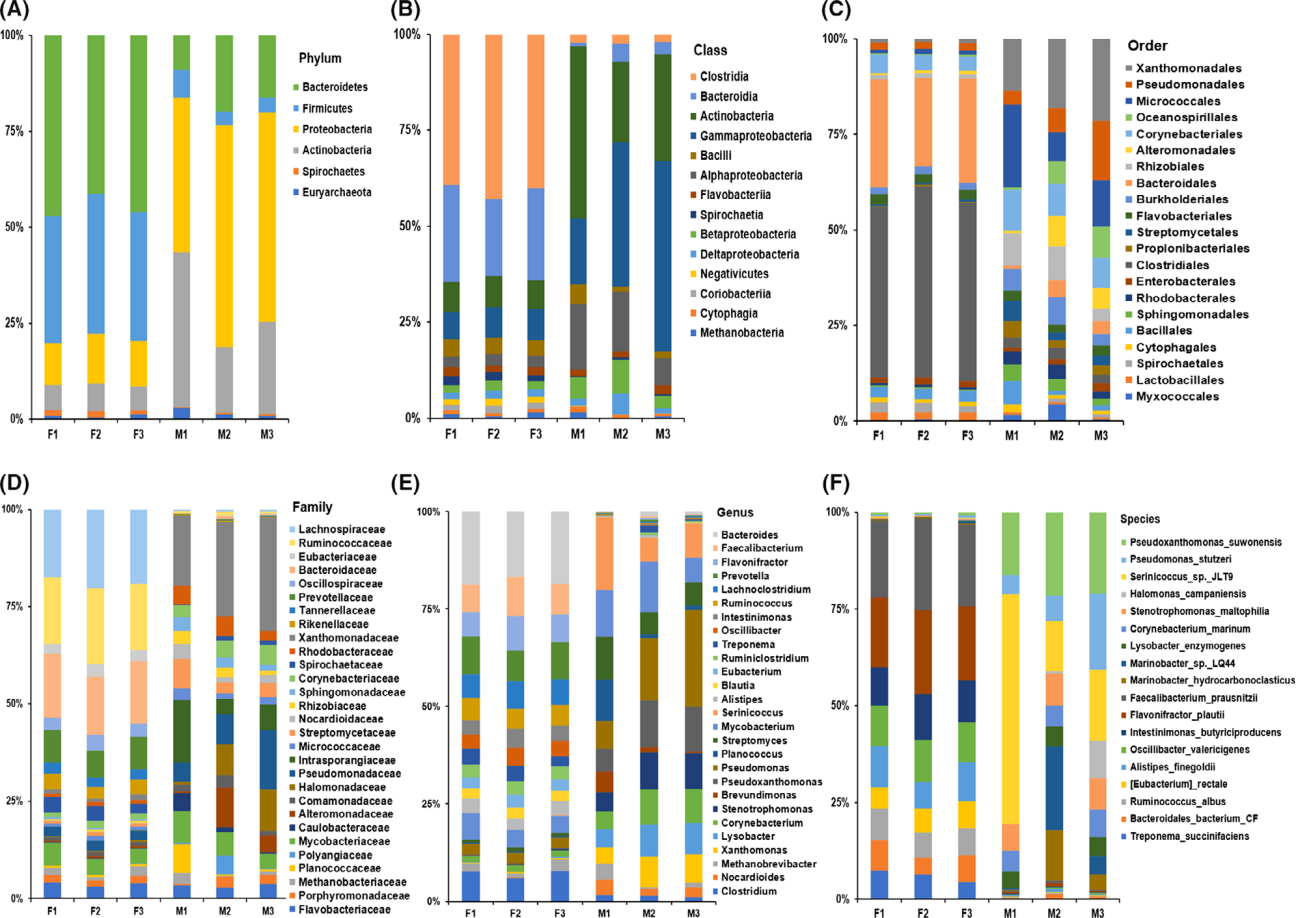
RAs of taxonomic composition in faecal samples. (RAs) ˃1%. (A) Phylum. (B) Class. (C) Order. (D) Family. (E) Genus. (F) Species.

The RAs of taxonomy were measured by assessing the relative number of species‐specific reads corresponding to queried reference sequences. The majority of species‐specific mapped sequence data could be attributed to a relatively small number of individual species. With the filter criterion RAs ˃ 1%, we obtained 6 phyla, 14 classes, 21 orders, 29 families, 27 genera and 18 species in faeces samples. The RAs (%) of microbiome composition were shown in Fig. 1 and Table S3. Despite the highly diverse bacterial communities between group F and group M, the microbial compositions of the fresh faeces were distinctively different from those of manure at every taxonomic level (Fig. 1, Tables [Link], [Link], [Link]).

**Fig. 2:**
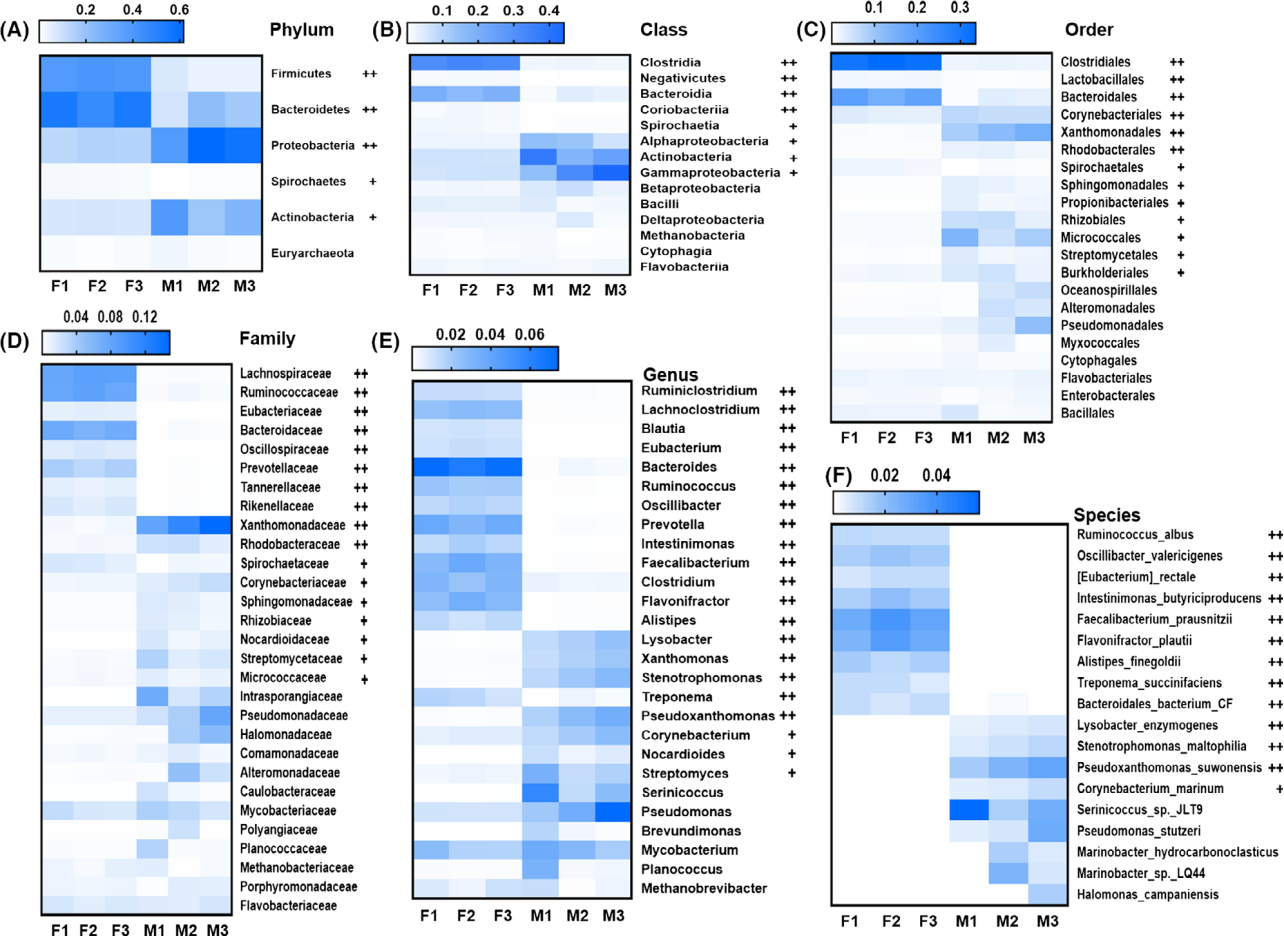
Heat map analysis of differential abundances of taxonomic annotations in M samples compared with those in F samples. (RAs) ˃1%, +=*P* < 0.05, ++= *P*< 0.01, higher abundance was indicated by ‘blue’, no significant difference is indicated by ‘white’. (A) Phylum. (B) Class. (C) Order. (D) Family. (E) Genus. (F) Species.

When comparing the abundance of the bacterial phylum, the RAs of *Proteobacteria* (11.96% vs. 50.91%) and *Actinobacteria* (6.7% vs. 27.14%) were highly enriched in manure compared with those in fresh faeces (*P* < 0.05). In contrast, the RAs of *Firmicutes* (34.37% vs. 4.86%) and *Bacteroidetes* (44.78% vs. 15.03%) were significantly decreased in manure compared with those in fresh faeces (*P *< 0.01) (Fig. 2A, Fig. S3A). A similar trend of significant change was found at the class level and order level; four significantly changed classes and orders were subordinated to four corresponding significantly changed phyla. At the class level, *Gammaproteobacteria* and *Actinobacteria* RAs were highly enriched in manure compared with those in fresh faeces (*P* < 0.05). RAs of *Clostridia* and *Bacteroidia* were significantly decreased in manure compared with those in fresh faeces (*P* < 0.01) (Fig. 2B, Fig. S3B). At the order level, RAs of *Xanthomonadales* and *Micrococcales* were highly enriched in manure compared with those in fresh faeces (*P* < 0.05). RAs of *Clostridiales* and *Bacteroidales* were decreased significantly in manure compared with those in fresh faeces (*P* < 0.01) (Fig. 2C, Fig. S3C).

There were 17 statistically significant differences between the F and M groups at the family level. The RAs of 8 of them were significantly decreased in manure compared with those in fresh faeces (*P* < 0.05), while the RAs of 9 of them were highly enriched in manure compared with those in fresh faeces (*P* < 0.05) (Fig. 2D, Fig. S3D). The microbial composition was also distinctly different at the genus level. Among the top 27 most abundant genera (RA ˃ 1%), the RAs of *Lysobacter*, *Pseudoxanthomonas*, *Nocardioides*, *Stenotrophomonas*, *Xanthomonas*, *Streptomyces* and *Corynebacterium* were highly enriched in manure compared with those in fresh faeces (*P* < 0.05). The RAs of *Treponema*, *Clostridium*, *Ruminiclostridium*, *Alistipes*, *Blautia*, *Lachnoclostridium*, *Bacteroides*, *Eubacterium*, *Prevotella*, *Ruminococcus*, *Oscillibacter*, *Flavonifractor*, *Intestinimonas* and *Faecalibacterium* were significantly decreased in manure compared with those in fresh faeces (*P* < 0.05) (Fig. 2E, Fig. S3E).

Collectively, these data indicated that there were highly diverse bacterial communities in these 6 faecal samples. Most importantly, the microbial composition was significantly changed during faecal accumulation.

### The diversity of pathogenic microorganisms in fresh faeces and manure

**Fig. 3:**
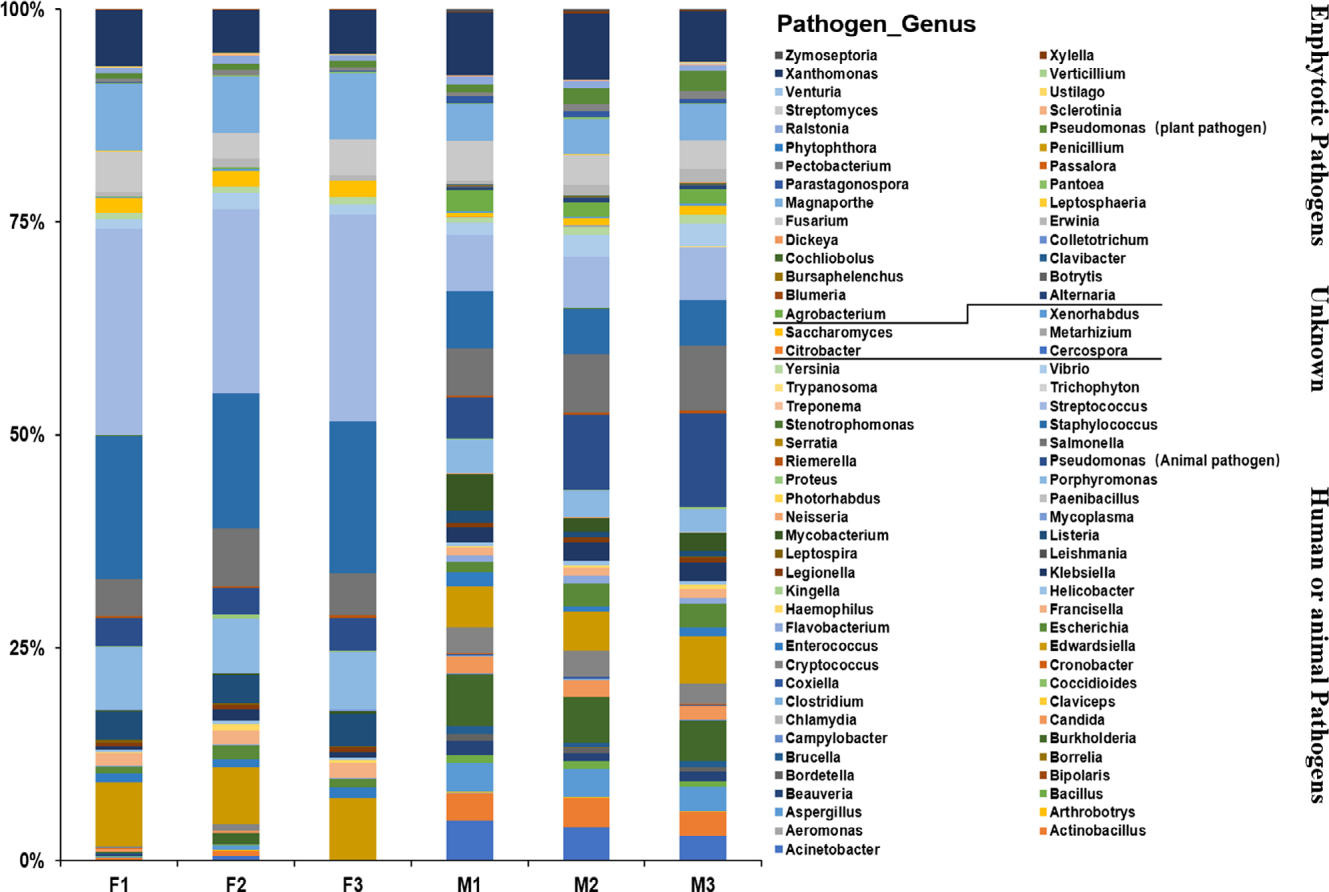
RAs of pathogenic genus annotation composition in fresh faeces (F) and manure (M) samples.

To obtain more in‐depth view of the pathogenic microbiota present within fresh faeces and manure in dairy farm, the PHI database was used to detect differentially abundant taxa of pathogenic bacteria, virulence genes and related diseases, using the default parameters. We obtained 87 pathogenic genera in fresh faeces and manure samples. Of these, 53 human and animal pathogenic genera, 29 enphytotic genera and 5 unknown pathogenic genera were identified (Fig. 3), suggesting that there were highly diverse pathogenic communities in faecal samples in dairy cow breeding farm.

**Fig. 4:**
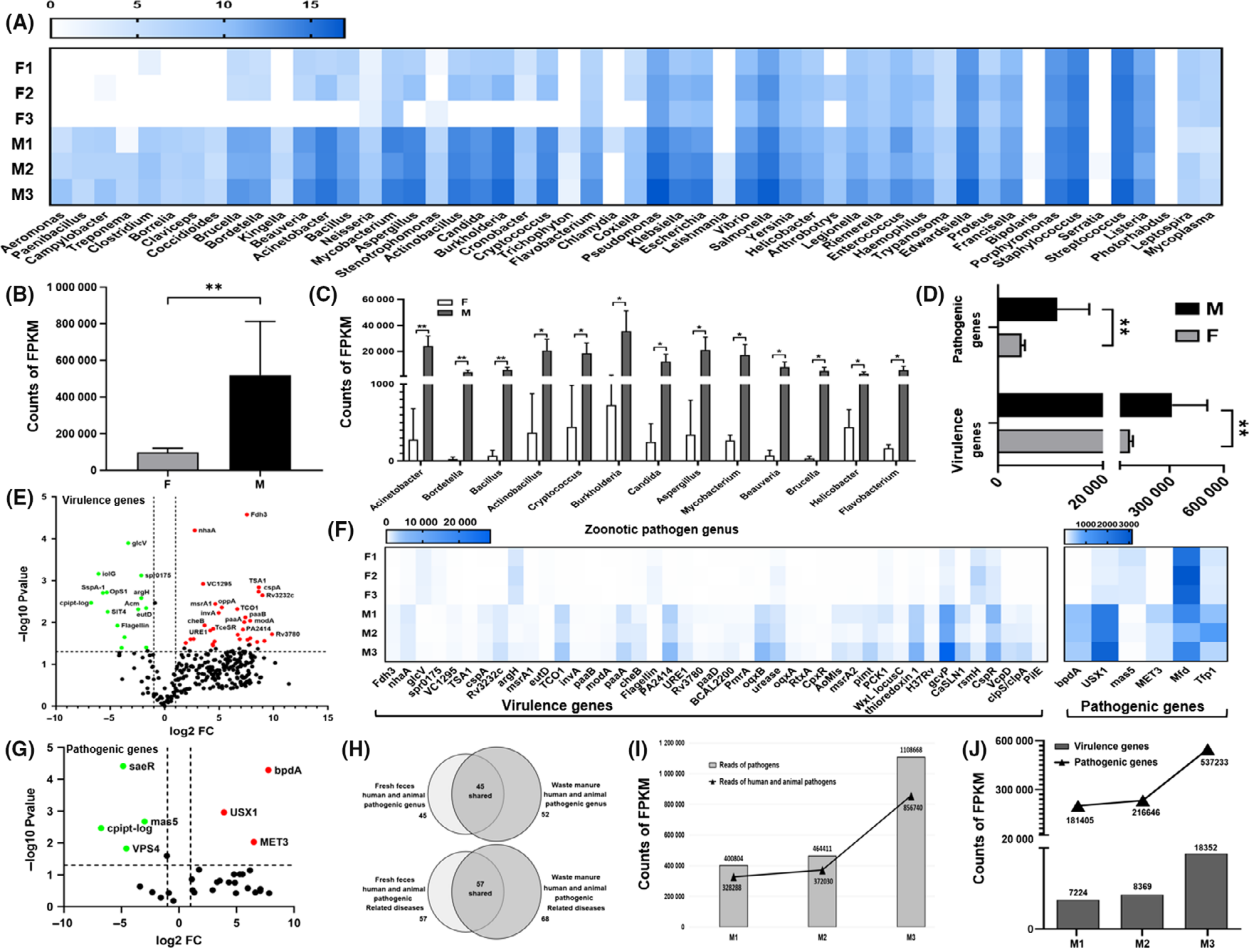
Abundance characterization of human and animal pathogenic genera and virulence genes in fresh faeces and manure samples. (A) Heat map analysis of differential logRAs of human and animal pathogenic genera. A higher abundance is indicated by ‘blue’, and no significant difference is indicated by ‘white’. (B) Total FPKM of pathogenic genera between group F and group M. (C) Column chart analysis of significantly different FPKM reads of the pathogenic genera between group F and group M. (RAs) ˃0.5%. (D&F) Column chart and heat map analysis of differential FPKM of virulence genes and pathogenic genes. (E&G) Volcano plot analysis of the differential abundance of virulence genes and pathogenic genes between group F and group M. (H) Number of differentially abundant human and animal pathogenic genera and related diseases in fresh faeces and manure. (I) FPKM of pathogens and human and animal pathogens enhanced in group M with increased manure stacking depth (M1‐5 cm, M2‐10 cm, M3‐15 cm). (J) FPKM of virulence genes and pathogenic genes enhanced in group M with increased manure stacking depth (M1‐5 cm, M2‐10 cm, M3‐15 cm). *P ˂ 0.05, **P ˂ 0.01.

After PHI database annotation, the RAs of 53 human and animal pathogenic genera were different between group F and group M (Fig. 4A, Table 1). Compared with group F, group M exhibited substantial enrichment in total FPKM reads of pathogenic genera (human and animal pathogenic and enphytotic genera) (*P* < 0.01) (Fig. 4B, Fig. S4C). With the filter criteria RAs ˃ 0.5% and *P* ˂ 0.05, we found the RAs of 13 human and animal pathogenic genera were significantly increased in manure compared with those in fresh faeces, namely, those of *Acinetobacter*, *Bordetella*, *Bacillus*, *Actinobacillus*, *Cryptococcus*, *Burkholderia*, *Candida*, *Aspergillus*, *Mycobacterium*, *Beauveria*, *Brucella*, *Helicobacter* and *Flavobacterium* (Fig. 4C). Meanwhile, the RAs of 6 enphytotic genera were significantly increased in manure samples under the same filter criteria (Fig. S4A,B). These data suggested that pathogenic microbes could proliferate when faeces accumulated to manure.

**Table 1:** Differential pathogenic genuera composition and related diseases in fresh faeces (F) and waste manure (M) samples.

Pathogen‐genus	Related diseases	Number of contigs(FPKM)						Fold change	*P*‐value
		F1	F2	F3	M1	M2	M3		
Human or animal pathogens									
*Acinetobacter*	Nosocomial infections	95	739	0	19 954	19 409	33 034	86.70	0.006
*Actinobacillus*	Lung infections, Bacteremia	168	946	0	14 311	17 272	30 593	55.76	0.015
*Aeromonas*	Intestinal and extraintestinal infections	0	1	0	34	77	737	424.00	0.282
*Arthrobotrys*	Nematode trapping fungus	0	238	0	511	387	610	6.31	0.014
*Aspergillus*	Aspergillosis, Invasive pulmonary aspergillosis	168	852	0	14 728	16 393	32 402	62.22	0.021
*Bacillus*	Gastrointestinal diseases and local and systemic infections, Anthrax	142	58	0	4446	4867	7918	85.73	0.007
*Beauveria*	White muscardine disease	132	87	0	6847	4567	12 325	107.90	0.027
*Bipolaris*	Spot blotch disease	0	0	0	1	2	0	3.00	0.158
*Bordetella*	Bordetella pertussis	36	46	0	3699	3143	5747	151.67	0.006
*Borrelia*	Lyme disease	1	0	0	204	150	117	235.50	0.003
*Brucella*	Brucellosis	57	41	0	4024	3045	8104	153.26	0.032
*Burkholderia*	Melioidosis, Cystic fibrosis	288	1894	0	26 460	26 664	53 557	48.87	0.018
*Campylobacter*	Gastroenteritis	1	2	0	226	136	828	297.50	0.143
*Candida*	candidiasis	268	476	0	8582	9566	18 647	49.39	0.020
*Chlamydia*	Pneumonitis; upper genital tract infections	0	0	0	26	0	0	26.00	0.374
*Claviceps*	Ergot	1	1	0	142	222	157	173.67	0.002
*Clostridium*	Enteric diseases	5	0	0	311	732	521	260.67	0.013
*Coccidioides*	Respiratory disease	0	0	0	62	37	69	168.00	0.004
*Coxiella*	Q fever	47	72	0	685	864	1056	21.71	0.002
*Cronobacter*	Meningitis; bacteremia	22	10	0	514	331	683	46.30	0.008
*Cryptococcus*	Cryptococcosis	287	1047	0	12 838	15 471	27 663	41.93	0.016
*Edwardsiella*	Enteric septicaemia, Haemorrhagic septicaemia	7612	10 420	8578	21 292	22 645	62 583	4.00	0.121
*Enterococcus*	Nosocomial infections, Endocarditis, Probiotic gut bacteria	1111	1540	1528	7091	3258	11 008	5.11	0.063
*Escherichia*	Infections, Avian colibacillosis	800	2556	1119	5641	13 363	31 708	11.33	0.118
*Flavobacterium*	Bacterial cold‐water disease	129	154	219	2918	4629	8947	32.79	0.041
*Francisella*	Ferric and ferrous iron acquisition	1465	2594	2000	4072	4448	11 637	3.33	0.131
*Haemophilus*	Glässers Disease	147	1033	406	836	1567	5625	5.06	0.229
*Helicobacter*	Gastric infections	248	693	378	1809	2480	4364	6.56	0.035
*Kingella*	Diseases of the skeletal system in children and infective endocarditis	0	0	0	38	72	33	143.00	0.018
*Klebsiella*	Urinary tract infections; nosocomial pneumonia; intra‐abdominal infections	460	2017	727	7651	10 765	24 887	13.51	0.066
*Legionella*	Legionnaires disease	436	830	687	2058	3090	7105	6.27	0.090
*Leishmania*	Leishmaniasis	0	1	1	1	13	18	10.67	0.119
*Leptospira*	Leptospirosis	246	286	108	23	178	141	0.53	0.236
*Listeria*	Listeriosis	3416	5173	4550	6293	2990	7756	1.30	0.435
*Mycobacterium*	Tuberculosis	215	242	346	19 184	8354	24 164	64.31	0.022
*Mycoplasma*	Contagious agalactia	133	127	161	21	41	67	0.31	0.005
*Neisseria*	Neisseria meningitidis	4	5	6	170	301	565	64.75	0.043
*Paenibacillus*	American foulbrood	0	0	0	181	141	72	394.00	0.015
*Photorhabdus*	Immunosuppression; septicaemia; subsequent death	0	0	0	0	1	0	1.00	0.374
*Porphyromonas*	Periodontitis	7407	9962	7922	16 788	15 272	30 820	2.49	0.067
*Proteus*	Urinary tract infections	101	729	185	439	877	2272	3.53	0.218
*Pseudomonas *(Animal pathogen)	Septicaemia, Infections	3376	4803	4589	21 262	43 872	12 5078	14.90	0.134
*Riemerella*	Fibrinous Serositis	231	280	351	776	1148	2850	5.53	0.111
*Salmonella*	Salmonellosis, Food poisoning	4449	10 585	5758	24 460	34 378	86 246	6.98	0.098
*Serratia*	Haemolymph bleeding	0	1	0	0	2	1	1.50	0.374
*Staphylococcus*	infections; food poisoning; respiratory diseases, Osteomyelitis	17 185	24 707	21 023	28 844	26 821	61 136	1.86	0.188
*Stenotrophomonas*	bacterial infection	2	1	4	87	187	216	61.25	0.015
*Streptococcus*	infection, Pneumococcal pneumonia, Streptococcosis	24 748	33 472	28 509	28 571	29 902	70 006	1.48	0.371
*Treponema*	Periodontitis	0	0	0	2	143	119	264.00	0.113
*Trichophyton*	Ringworm	0	0	0	27	6	4	37.00	0.169
*Trypanosoma*	Chagas disease	43	46	44	193	132	269	4.43	0.018
*Vibrio*	Cholera	1163	3107	1414	6277	13 015	30 876	8.82	0.114
*Yersinia*	Gut‐associated diseases, Pneumonic plague	697	1103	997	2678	4604	11 399	6.68	0.116
*Cercospora*	unknown	3	1	0	15	3	8	5.20	0.111
*Citrobacter*	unknown	1	1	0	233	90	127	150.00	0.025
*Metarhizium*	unknown	15	8	10	196	909	142	36.68	0.177
*Saccharomyces*	unknown	1819	2771	2211	1960	3812	10 976	2.46	0.296
*Xenorhabdus*	unknown	70	332	88	524	1204	3986	11.64	0.177
Enphytotic pathogens									
*Agrobacterium*	Crown gall disease	90	364	0	11 185	8328	19 101	84.87	0.017
*Alternaria*	Brown spot disease	9	5	0	1242	2423	4271	529.07	0.040
*Blumeria*	Wheat powdery mildew	0	0	0	20	9	86	115.00	0.186
*Botrytis*	Grey mould	21	47	0	834	694	1720	47.07	0.030
*Bursaphelenchus*	Pine wilt disease	0	0	0	180	277	358	815.00	0.006
*Clavibacter*	Wilt and canker disease of tomato	28	0	0	542	218	455	41.90	0.015
*Cochliobolus*	Southern corn leaf blight	0	0	0	147	225	770	1142.00	0.124
*Colletotrichum*	Anthracnose	0	0	0	117	268	539	924.00	0.067
*Dickeya*	Soft rot disease	0	0	0	30	100	198	328.00	0.088
*Erwinia*	Fire blight	452	1522	685	1580	6103	18 242	9.75	0.195
*Fusarium*	Fusarium ear blight	4943	4611	4950	20 093	17 685	37 842	5.21	0.033
*Leptosphaeria*	Phoma stem canker	59	51	45	205	207	134	3.50	0.006
*Magnaporthe*	Rice blast	8041	10 328	9091	18 561	20 814	48 635	3.20	0.106
*Pantoea*	Stewart’s wilt	115	183	198	640	754	937	4.69	0.002
*Parastagonospora*	Glume blotch	67	63	103	3560	3570	5708	54.86	0.004
*Passalora*	Leaf mould	0	0	1	35	6	11	26.00	0.131
*Pectobacterium*	Rotting of tubers; blackleg disease of the plant stem	449	901	583	1947	4433	10 640	8.80	0.124
*Penicillium*	Blue mould	0	0	0	1	0	1	2.00	0.116
*Phytophthora*	Soya bean root rot	0	0	1	0	0	0	0.00	0.374
*Pseudomonas *（plant pathogen）	Bacterial leaf spot, Bacterial speck of tomato	592	1213	845	3965	9018	26 044	14.72	0.144
*Ralstonia*	Bacterial wilt	675	1449	740	4098	4536	7784	5.73	0.019
*Sclerotinia*	White mould/stem rot	38	261	79	626	626	1129	6.28	0.021
*Streptomyces*	Common scab of potato	0	0	0	3	15	1977	1995.00	0.368
*Ustilago*	Ustilago maydis	119	123	138	327	187	776	3.39	0.163
*Venturia*	Apple scab	0	0	0	1	2	13	16.00	0.238
*Verticillium*	Wilt diseases	0	0	0	0	4	0	4.00	0.374
*Xanthomonas*	Bacterial leaf blight, Rice bacterial blight	6756	7918	5982	31 744	38 910	67 521	6.69	0.023
*Xylella*	Pierce disease of grapes and citrus variegated chlorosis	69	130	135	505	1446	1482	10.25	0.032
*Zymoseptoria*	septoria speckled leaf blotch	5	1	4	1081	804	1161	276.91	0.001

We analysed the RAs of pathogenic genes and virulence genes in all samples to investigate whether the pathogenic microbes were enriched after faeces accumulation. Compared with group F, group M exhibited substantial enrichment of total FPKM reads of human and animal pathogenic virulence and pathogenic genes (*P* < 0.01) (Fig. 4D). With the filter criteria RAs ˃ 0.5%, pathogenic genes and virulence genes were related to Pathogen‐genus and the results list in Table 1, a total of 307 virulence genes and 32 pathogenic genes were found to be differentially expressed between group F and group M, of which 43 virulence genes and 6 pathogenic genes were significantly differentially expressed (Fig. 4E, F, G). There were 22 pathogenic genes (51.16%) and 232 virulence genes (75.57%) upregulated in manure compared with their expression in fresh faeces, of which 3 pathogenic genes and 40 virulence genes were upregulated significantly (Fig. 4E,G). Similarly, analysis of enphytotic virulence and pathogenic genes showed the same result (Fig. S4D, F, G). These data indicated that pathogenicity and virulence genes will be enriched with the enrichment of pathogens when faeces accumulate to manure.

Additionally, we found that the genera (M vs. F = 52 vs. 45) and the related diseases (M vs. F = 68 vs. 57) of human and animal pathogens in manure were higher than in fresh faeces (Fig. 4H). These results suggested that the pathogens of the cow breeding farm environment could increase their harmfulness in manure through proliferating usage of fresh faeces as a medium. Moreover, the RAs of human and animal pathogens, including *Enterococcus*, *Klebsiella*, *Porphyromonas*, *Legionella*, *Salmonella*, *Vibrio*, *Yersinia*, *Escherichia*, *Edwardsiella*, *Pseudomonas* and *Streptococcus,* were enriched in manure compared with those in fresh faeces, but the P‐value of these changes was not significant (Table 1). We found the FPKM of pathogens, human and animal pathogens, virulence genes and pathogenic genes enhanced in group M with increased manure stacking depth (M3‐15 cm ˃ M2‐10 cm ˃ M1‐5 cm) (Fig. 4I,J). These results indicated that manure stacking depth could influence the enrichment of pathogens.

### Characterization of antibiotic resistance in fresh faeces and manure

**Fig. 5:**
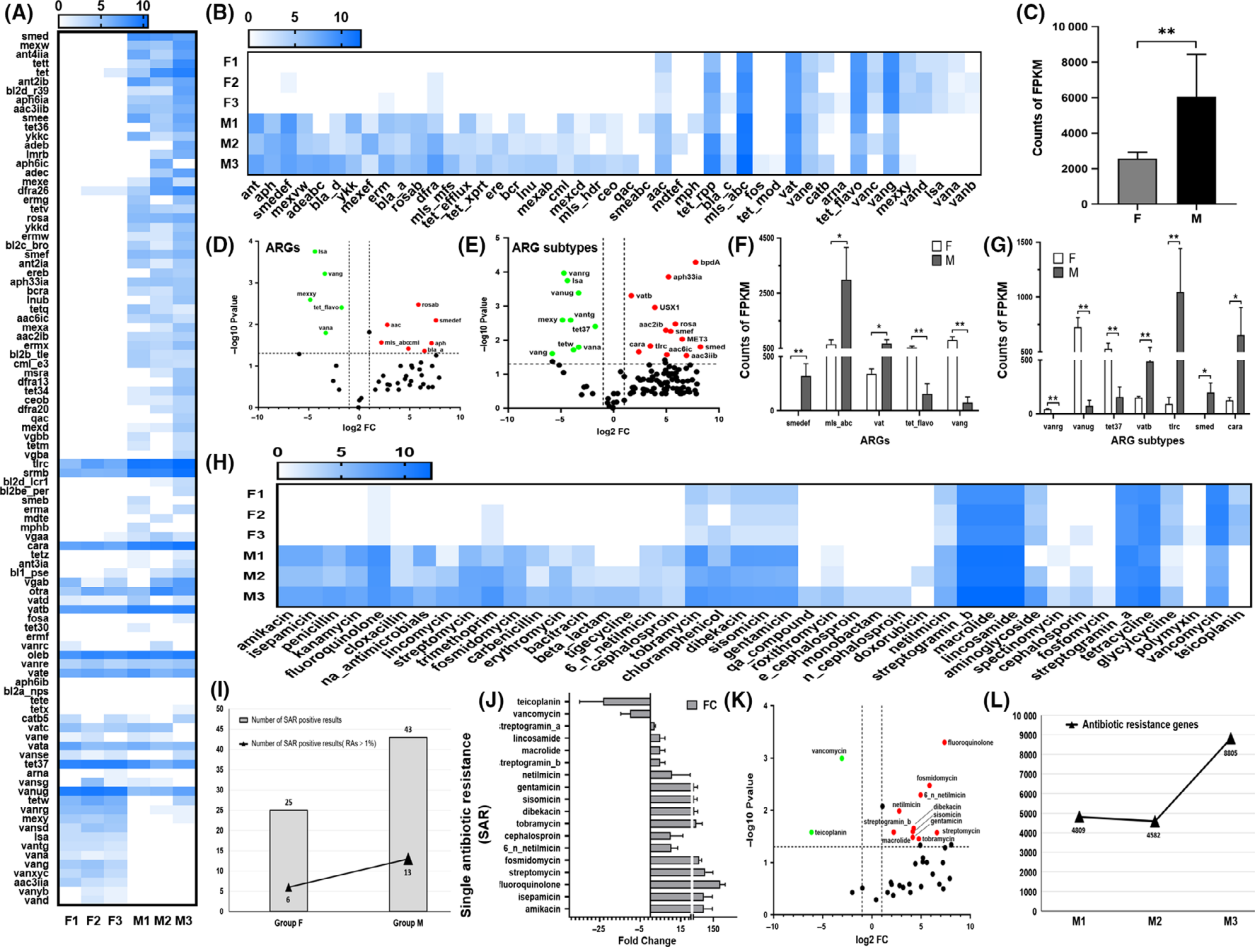
RA characterization of antibiotic resistance genes (ARGs) and types in fresh faeces and manure samples. (A&B) Heat map analysis of differential abundances of ARGs and ARG subtypes. (C) Total FPKM of ARGs between group F and group M. (D&E) Volcano plot analysis of the difference in ARGs and ARG subtypes between group F and group M. (F&G) Column chart analysis of significantly different FPKM of ARGs and ARG subtypes between group F and group M. (H&J) Heat map analysis of differential RAs of single antibiotic resistance (SAR). (I) Number of SAR‐positive results and significant positive results (RAs˃1%) between group F and group M. (K) Volcano plot analysis of the differential abundance of an SAR between group F and group M. (L) FPKM of ARGs enhanced in group M with increased manure stacking depth (M1‐5 cm, M2‐10 cm, M3‐15 cm). *,+=*P *< 0.05, **,++=*P *< 0.01.

To investigate ARGs in fresh faeces and manure, assembled genes were clustered and then analysed for the abundance of ARGs, ARG subtypes and antibiotic resistance drug types between group F and group M (Table 2, Tables S5,S6). In total, 45 ARGs and 96 ARG subtypes were differentially expressed in all samples (Table S5, Fig. 5A,B). ARGs were found with higher abundance (6065.33 ± 2375.33 vs. 2566.33 ± 355.87, *P* < 0.05) in manure than in fresh faeces (Fig. 5C). Compared with group F, group M exhibited upregulation of 35 ARGs (77.78%) and 71 ARG subtypes (68.93%) (Table S5). These results suggested that the overall level of antibiotic resistance was enhanced in the process of faecal accumulation.

**Table 2:** Significant differential number of contigs(FPKM) in single antibiotic resistance(SAR) between Group F and Group M

Antibiotic	Number of contigs (FPKM)						Fold change	*P*‐value
	F1	F2	F3	M1	M2	M3		
Fluoroquinolone	2	2	2	462	364	339	166.4286	0.001
Vancomycin	780	1029	785	62	81	169	0.120231	0.001
Fosmidomycin	1	0	1	46	52	76	58	0.003
6_n_netilmicin	0	0	0	9	8	14	31	0.005
Streptogramin_a	441	325	328	825	620	855	2.100457	0.008
Netilmicin	16	6	4	53	48	83	6.814815	0.010
Dibekacin	16	7	4	236	86	208	18.92857	0.022
Sisomicin	16	7	4	227	78	194	17.82143	0.026
Macrolide	483	838	597	2425	2179	4288	4.633663	0.026
Lincosamide	483	838	597	2418	2179	4288	4.630016	0.026
Teicoplanin	11	34	25	0	1	0	0.014085	0.026
Streptogramin_b	483	838	597	2424	2180	4306	4.643043	0.027
Streptomycin	1	0	1	41	129	123	97.66667	0.029
Gentamicin	16	7	4	226	69	202	17.75	0.033
Tobramycin	16	7	4	332	98	335	27.32143	0.035
Amikacin	0	0	0	106	29	133	268	0.046
Isepamicin	0	0	0	106	29	133	268	0.046
Cephalosproin	0	0	0	17	6	7	30	0.047

With the filter criteria fold change ˃ 2 and *P*‐value <0.05, we obtained 11 ARGs and 22 ARG subtypes that were differentially expressed between group F and group M (Fig. 5D,E). The RAs of *tlrc*(mls_abc), *cara*(mls_abc), *smed*(smedef) and *vatb*(vat) were enhanced after faecal accumulation (RA ˃ 1%, *P* < 0.05). Furthermore, the RAs of *vanrg*(vang), *vanug*(vang) and *tet37*(tet_flavo) were reduced during faecal accumulation (RAs ˃ 1%, *P* < 0.05) (Fig. 5F,G). It is interesting that all significantly different ARGs except *smed*(smedef) were found with higher RAs in fresh faeces than in manure and then enhanced or reduced after faecal accumulation. The increased RAs of smedef was much lower in group F (0.05 ± 0.02%) and significantly higher in group M (5.78 ± 3.19%), with its own characteristics. The results demonstrated that there was a large number of ARGs in cow faeces on the breeding farm; a fraction of ARGs decreased after faecal accumulation, but most of the ARGs increased during the faecal accumulation process, including the ones that were present in the fresh faeces and exogenous sources or the ones newly produced during faecal accumulation.

Subsequently, we annotated single antibiotic resistance (SAR) using ARGs. A total of 43 SARs were obtained with differential RAs between group F and group M (Fig. 5H). There were 25 (6/25 RAs ˃ 1%) positive results of SAR in fresh faeces and 43 (13/43 RAs ˃ 1%) positive results of SAR in manure (Fig. 5I). Compared with those in group F, RAs of 18 SAR were significantly increased and of 2 SAR were significantly decreased in group M (Fig. 5J). The SAR of vancomycin was with the highest number of reads and decreased level during faecal accumulation. Macrolide, lincosamide and streptogramin_b had the highest number of reads (both in group F and group M) and increased levels during faecal accumulation (Fig. 5K, Table 2). We also found 13 SARs that were much lower in group F and significantly higher in group M, such as the ARG smedef (Table 2). Multidrug antibiotic resistance analysis indicated that the type lincosamide/macrolide/streptogramin_b had the highest increased level during faecal accumulation (Table [Link], [Link]). These data indicated that the process of faecal accumulation could resulted in significantly increased microbial antibiotic resistance.

Interestingly, we also found FPKM of ARGs enhanced in group M with increased manure stacking depth (M3‐15 cm ˃ M2‐10 cm ˃ M1‐5 cm, Fig. 5L), similar to the result for pathogens in group M (Fig. 4I,J). The results lend further credence to the hypothesis that manure stacking depth could influence the enrichment of antibiotic resistance.

## Discussion

Microbiome studies described the significance of the microbial community that was associated with the host organism (Brooks *et al.*, 2017). However, less than 1% of all microbial species can be cultured in vivo (Locey and Lennon, 2016). Metagenomics, which a method could directly analyses the total DNA from environmental samples, provides a powerful strategy for unveiling novel microbes in microbial communities without the technical challenges of cultivation (Doane *et al.*, 2017). In this study, we analysed the metagenomic data from three fresh faeces and three manure samples in a dairy cow breeding farm in Ningxia. Ningxia is one of the top ten pastoral areas in China. We found highly diverse and distinctively different bacterial communities between fresh faeces and manure. Present studies agree that microbial diversity is a major feature in fresh faeces or composted samples (Kim *et al.*, 2018). We and others found that the dominant phyla of the community structure in cow faeces were *Firmicutes, Proteobacteria, Bacteroidetes* and *Actinobacteria* (Wang *et al.*, 2017). Furthermore, we found that the RAs of *Proteobacteria* (11.96% vs. 50.91%) and *Actinobacteria* (6.7% vs. 27.14%) were highly enriched, while those of *Firmicutes* (34.37% vs. 4.86%) and *Bacteroidetes* (44.78% vs. 15.03%) were significantly decreased during faeces accumulation, with a similar trend of change at the class and order levels. With the annotation, the main enriched microbial species were aerobic or facultative anaerobic microorganisms during faeces accumulation, such as *Alphaproteobacteria* and *Gammaproteobacteria*, while the reduced microbial species were always anaerobic microorganisms, such as *Bacteroidia* and *Clostridia*. However, only a few publications have reported microbial communities in fresh faeces accumulating to manure under natural conditions. We believe that the process of faecal accumulation does more harm than good.

Our metagenomic data showed highly diverse pathogenic communities in faecal samples from the dairy cow breeding farm, the results were consistent with other studies (Kim *et al.*, 2018; Muñoz‐Vargas *et al*., 2018). Previous research has shown that the largest number of pathogen genes was detected in dairy waste and they may come from farm environments or other wastes, which was a reminder of the potential risk to human health presented by the farm environment and consumption of unpasteurized milk (Sledz *et al.*, 2017). We found the species and RAs of human and animal pathogens were significantly increased during faecal accumulation. There were 52 human and animal pathogenic genera in manure compared with 45 kinds in fresh faeces. Of these, the RAs of 13 human and animal pathogenic genera were significantly increased in manure compared with those in fresh faeces (Fig. 4C). The results indicated that faecal accumulation could be regarded as a process of the enrichment of pathogens in faeces and the environment during the use of manure as the medium. It was likely resulted from that microbial pathogens could benefited from the rich source of available nutrients in organic manures like nitrogen, phosphorus, potassium, sodium, copper, zinc, calcium, selenium, manganese, magnesium and sulphur. (Sledz *et al.*, 2017). At the same time, recent research indicated that stream water and rain in dairy breeding farms could help manure mix with water and expand the spread of pathogens (Haack *et al.*, 2016). Therefore, faecal accumulation is the one of the major sources of pathogens in dairy farm environments. We analysed the human and animal pathogenic pathogens only at the genus level, despite the PHI database annotation results at the species level. These results have been sufficient to offer the potential to address both the source of and risks associated with faecal pollution, for example, the potential pathogenicity of *Acinetobacter* in nosocomial infections (Almasaudi, 2018), *Bacillus* in anthrax (Welkos *et al.*, 2015) and *Actinobacillus* in bacteremia (Cosford, 2018). Additionally, we found that the RAs of 6 enphytotic genera were also significantly increased in manure samples, namely, *Fusarium* (Fusarium ear blight, Fusarium head blight), *Xanthomonas* (bacterial leaf blight), *Parastagonospora* (glume blotch), *Agrobacterium* (crown gall disease), *Alternaria* (brown spot disease) and *Ralstonia* (bacterial wilt). This result suggests that pathogens from manure could spread to the plant environment and enphytotic pathogens could affect the yield and quality of crops when manure is used as a fertilizer. Thus, complex environments enhance bacterial community interactions and metabolism; These results being consistent with previous studies (Hãger et al., 2016; Sledz *et al.*, 2017).

Pathogens always harm the host through virulence genes. In this study, we found that three pathogenic genes and 40 virulence genes from human and animal pathogenic genera were significantly upregulated in manure compared with their expression in fresh faeces. Among them, the top 10 virulence genes and three pathogenic genes included the *USX1*, *MET3*, *URE1* and *Tco1* genes produced from *Cryptococcus neoformans,* which may cause cryptococcosis disease in either humans or animals (Moyrand *et al.*, 2002; Pascon *et al.*, 2004; Feder *et al.*, 2015; Kong *et al.*, 2017); the *bpdA* and *CspA* genes producing from *Brucella melitensis* may cause ovine brucellosis in animals (Zhang, 2018); the *nhaA* and *CheA* genes produced by *E. coli*, which may cause meningitis or infect humans or animals (Tavaddod and Naderi‐Manesh, 2016); the *Fdh3* gene produced by *Candida albicans*, which may cause disseminated candidiasis in either humans or animals (Tillmann *et al.*, 2015); the *oqxB* gene produced by *Klebsiella pneumoniae*, which may cause pneumonia disease in animals (Nicolas‐Chanoine *et al.*, 2018); the *VC1295* gene produced by *Vibrio cholerae*, which may cause cholera disease in either humans or animals (Conner *et al.*, 2017); the *RV3232c* gene associated with *Mycobacterium tuberculosis*, which may cause tuberculosis in either humans or animals (Singh *et al.*, 2016); and the *PA2414* gene associated with *Pseudomonas aeruginosa* which may cause nosocomial infections disease in either humans or animals (Dubern *et al.*, 2015). The results for the virulence genes were consistent with those for pathogenic bacteria. Interestingly, we found FPKM of pathogens, human and animal pathogens, virulence genes and pathogenic genes enhanced in group M with increased manure stacking depth (M3‐15 cm ˃ M2‐10 cm ˃ M1‐5 cm), which made some human and animal pathogens have higher RAs and be enriched in manure compared with those in fresh faeces, but the *P*‐value of these changes was not significant. Such species included *Pseudomonas, Streptococcus* and *Escherichia,* which may infect either humans or animals. We believe that these pathogens are noteworthy and need to be analysed in a subsequent validation utilizing a larger cohort.

Overuse of antibiotics in animal husbandry is a worldwide problem (Ferri *et al.*, 2017). China is one of the largest consumers of antibiotics worldwide (Zhang *et al.*, 2015). In the present study, we found that the overall level of RAs of ARGs and ARG subtypes was increased during manure accumulation. In total, 11 ARGs and 22 ARG subtypes were enhanced in manure. Among them, *tlrc*(mls_abc), *cara*(mls_abc), and *vatb*(vat) were enhanced during faecal accumulation, with increased RAs in either fresh faeces or manure, while *vanrg*(vang), *vanug*(vang) and *tet37*(tet_flavo) were reduced in the process, with higher RAs in either fresh faeces or manure. This result means that the resistances to streptogramin_a (*vat*), lincosamide/macrolide/streptogramin_b (*mls_abc*), vancomycin (*vang*) and tetracycline (*tet_flavo*) were found with high RAs in fresh faeces. Previous studies reported that lincosamide/macrolide/streptogramin_b was commonly used in livestock breeding as feed additives to promote animal growth and maturity (Hao *et al.*, 2015). In addition, vancomycin and tetracycline are commonly used in bacteriostasis and sterilization in dairy breeding farms (Holmes *et al.*, 2018). Consistent with relevant studies, ARGs for tetracyclines were found in the manure of chickens, pigs and cows (Mitchell *et al.*, 2015). Previous study indicated that tetracyclines, vancomycin and streptogramin_b should decrease in vivo after feeding of livestock or in vitro during faeces composting (Selvam *et al.*, 2013; Mitchell *et al.*, 2015). However, we found degradation of only vancomycin, with no change in tetracycline, but increased levels of lincosamide, macrolide, streptogramin_b and streptogramin_a. Moreover, fluoroquinolone resistance (smedef gene) was not found in fresh faeces but increased significantly in manure, speculating that the accumulated manure was also the transfer station of ARGs to enrich the ARGs in the environment. One of the primary causes of enrichment of ARGs is HGT which could make ARGs vestigial even if the bacteria carrying ARGs die (Wang *et al.*, 2018). We observed RAs of microbes and pathogenic microbe enrichment when faeces accumulated to manure. With HGT, ARGs transferred from the host bacteria to other bacteria, pathogenic microbes, even host and the environment. Eventually, humans could become the ultimate receptors of ARGs. Previous studies have also confirmed that HGT of ARGs could impact food safety by the enrichment of ARGs in the pasture or in the composting process of pig, cow and poultry manure (Soucy *et al.*, 2015; Baker *et al.*, 2016; Pornsukarom and Thakur, 2017).

Interestingly, consistent with the results of virulence genes and pathogens being enhanced in group M with increased manure stacking depth (M3‐15 cm ˃ M2‐10 cm ˃ M1‐5 cm), a similar trend was found in ARGs in manure. It is not clear whether the changes in waste manure with stacking depth were caused by the time, local environment, or microbial interactions during accumulation. We will pay more attention to these questions by an in‐depth examination.

In summary, through metagenomic analysis of fresh faeces and manure samples in a dairy farm in Ningxia, we provided evidence that (i) manure accumulation could significantly increase the abundances of the microbial and pathogenic constituents (ii) levels of virulence genes and ARGs increased with the enrichment of microbes and pathogens when faeces accumulated to manure and (iii) manure accumulation and usage without effective manure management is an unreasonable approach that could enrich pathogenic microorganisms and ARGs in the environment. Our findings provide insights into the influence of animal agriculture on manure accumulation and pollution in the breeding farm environment. Additional research of this type and a scale up would lend improved insight into the overall influence and interaction of animal waste on water, soil and other areas impacted by faecal accumulation and the factors that influence pathogen occurrence in products from dairy cows.

## Experimental procedures

### Specimens

A total of three fresh faeces and three manure specimens were collected in a scaled dairy cow breeding farm (Breeding stock:1500) in Ningxia, China (N38°20′, E106°16′). Each fresh faecal sample was a mixture from randomly chosen fresh faeces of 10 cows. A total of three fresh faecal samples were collected from different sectors in the same dairy cow farm in May 2017. Fresh faeces from 30 cows were collected to make the three fresh faecal samples. Manure sample was a mixture of the manure randomly collected from piled up manure; the specimens were mixtures collected at three different depths (5, 10 and 15 cm), and each was mixed in six sites in manure piled area (the faeces from all dairy cows in the breeding farm) after manure piled up for about 2 months in July 2017. The specimens were sealed in a 50 ml sterilized tube and then directly transported at 4℃ to the Key Laboratory of the Ministry of Education for Protection and Utilization of Special Biological Resources in western China, within 4 h. The specimens were stored in −80℃ for further use. The study protocol was reviewed and approved by the ethics committees of Ningxia University (Ningxia, China).

### DNA extraction and metagenome sequencing

DNA was extracted from 200 mg of samples with a QIAamp DNA Stool mini kit (QIAGEN, Dusseldorf, Germany) following the manufacturer’s instructions. DNA samples were quantified using NanoDrop 2000 (Thermo Scientific, Waltham, MA, USA) and confirmed using 0.8% agarose gel electrophoresis. Extracted DNA from each sample was stored at –20°C until use. The qualified DNA samples were broken into DNA fragments with a length of approximately 350 base pairs. After end repair, sequencing adaptor addition, purification and other steps, library construction from the samples were achieved using a TruSeq® Nano DNA LT Sample Prep Kit (Illumina, San Diego, ​CA, USA). The libraries had an insert size of 350 bp for every sample. Each library was sequenced by Illumina HiSeq 2000 equipment (Illumina). Metagenome sequencing were used (i) to determine the microbial diversity composition in fresh faeces and manure; (ii) to determine the diversity in the pathogenic community and antibiotic resistance during manure accumulation; and (iii) to evaluate the pathogen risk of natural manure accumulation and usage without effective manure management.

### Metagenome assembly and species annotation

An overview of the workflow developed with the tools applied at each step were list in Fig. S1. Raw sequencing reads were processed to obtain quality‐filtered reads for further analysis. First, sequencing adaptors were removed from sequencing reads using Cutadapt (Grassmann, 2019). Second, low‐quality reads were trimmed by using a sliding‐window algorithm (Wang *et al.*, 2015). Third, reads were aligned to the host genome using BWA (http://bio-bwa.sourceforge.net/) to remove host contamination (Li and Durbin, 2009). The following criteria were used for quality control: (i) reads were removed if they contained more than 3 N bases or more than 50 bases with low quality (<Q20), and (ii) reads were trimmed at the end with low quality (<Q20) or assigned as N. Once quality‐filtered reads were obtained, they were assembled to construct a metagenome for each sample by SOAP denovo software with the De Bruijn graph and overlap‐layout‐consensus (OLC) methods (Ye and Tang, 2016; Stewart *et al.*, 2018). Contig were classified using k‐mers and coverage. Gene abundance in each sample was estimated by soap.coverage (http://soap. genomics.org.cn/) based on fragments per kilobase per million mapped reads (FPKM) (Koch *et al.*, 2018). Taxonomy of every contig (gene annotation and amino acid annotation) was obtained by aligning them against the National Center for Biotechnology Information‐NR(NCBI‐NR) database (bacteria, fungi, archaea and viruses, e value ≤ 1E‐5). Taxonomic annotation (kingdom, phylum, class, order, family, genus and species) in each sample was estimated by taxonomy annotation results, and every contig was considered an organism. The RAs of a species in a sample were denoted as the sum of the genetic abundance of that species.

### Pathogenicity and antibiotic resistance analysis

The functional profiles of the nonredundant genes were obtained by annotation against the Gene Ontology (GO) and Kyoto Encyclopaedia of Genes and Genomes (KEGG) databases by using the DIAMOND alignment algorithm (Huson *et al.*, 2017). Based on the taxonomic and functional profiles of nonredundant genes, the Pathogen Host Interactions database (PHI‐base, http://www.phi-base.org/) was utilized to detect differentially abundant taxa (default parameters) of pathogenic bacteria, virulence genes and related diseases in every sample (Urban *et al.*, 2017). The Antibiotic Resistance Genes Database (ARDB) was used to detect differentially abundant taxa with ARGs and antibiotic‐resistant types in every sample, using the default parameters yy (Yang *et al.*, 2016). Gene abundance in each sample was estimated by soap.coverage based on FPKM. Species variation and functional gene variation in microbial communities across samples was analysed using Bray–Curtis distance metric principal coordinates analysis (PCoA). A heat map was analysed using Cluster 3.0 software. A volcano plot was analysed using ggPlot2 software (R).

### Statistical analysis

The abundances of assembled contigs were calculated based on the number of FPKM by using SPSS23.0 software (SPSS Inc. IBM, Chicago, IL, USA) and GraphPad Prism version 8.0.1 (GraphPad Software, San Diego, ​CA, USA). The data were presented as the mean ± Standard error of the mean (SEM) unless otherwise stated. Continuous variables were tested using a *t*‐test. All P‐values were two‐tailed, and the differences were considered significant if *P* < 0.05* or *P* < 0.01**.

## Conflict of interest

This work does not have any relationships with business‐related issues, and no conflicts of interest exist in the submission of this manuscript.

## Ethical approval

The study protocol was reviewed and approved by ethics committees of Ning Xia University (Ning Xia, China). The manuscripts reporting studies involving none of human participants, human data or human tissue.

## Consent for publication

I would like to declare on behalf of my co‐authors that the work described was original research that has not been published previously, and not under consideration for publication elsewhere, in whole or in part. The manuscript is approved by all authors for publication.

## Author contribution

Guangcun DENG: designed project, revised article and coordinated all aspects of work; Xiaoling WU: designed project, revised article, technical guidance; Xu ZHANG: participated in all experiment, data analysis, create figures, wrote article; Chenjie MA: NCBI‐NR database and taxonomic annotation, create tables; Wen ZHANG: PHI database analysis, data Cleansing; Wu LI: ARDB database analysis, data Cleansing; Jialin YU: specimens collected, sample preparation, documentation; Di XUE: Metagenome sequencing and analysing technical guidance, data quality control.

## Supporting information


**Fig. S1**. Flow chart of metagenomic sequencing and analysis at each step.

**Fig. S2**. Quality control and general characteristics of metagenomic sequencing results. (A) Assembly sequence length distribution; (B) species annotation krona frequency distribution; (C) Bray‐Curtis distance of RA data collected on fresh faeces and manure.

**Fig. S3**. Column chart analysis of differential RAs of taxonomic annotation between group F and group M. (RAs) ˃1%, *=* P *< 0.05, **=* P *< 0.01. (A) Phylum. (B) Class. (C) Order. (D) Family. (E) Genus. (F) Species.

**Fig. S4**. Comparison of enphytotic genera and virulence gene abundance in fresh faeces and manure samples. (A) Heat map analysis of differential RAs of the enphytotic genus annotation of every sample. A higher abundance is indicated by ‘blue’, and no significant difference is indicated by ‘white’. (B) Column chart analysis of significantly different FPKM of the enphytotic genera between group F and group M. (C) Total FPKM reads of the enphytotic genera between group F and group M. (D&E) Column chart and heat map analysis of differential FPKM of enphytotic virulence genes and pathogenic genes. (F&G) Volcano plot analysis of the differential abundance of enphytotic virulence genes and pathogenic genes between group F and group M. (H) FPKM of enphytotic pathogens, virulence genes and pathogenic genes enhanced in group M with increased manure stacking depth (M1‐5 cm, M2‐10 cm, M3‐15 cm). *,+=*P *< 0.05, **,++=* P *< 0.01.

**Table S1**. Data filtering and statistical analysis of metagenomic sequencing.

**Table S2**. The number of microbiome constituents at every taxonomic level between group F and group M.

**Table S3**. The RAs˃1% of microbiome constituents in fresh faeces (F) and manure (M) samples.

**Table S4**. Statistical analysis of the difference in taxonomic composition between group F and group M.

**Table S5**. Differential number of contigs (FPKM) in ARGs and ARG subtypes between group F and group M.

**Table S6**. Differential number of contigs (FPKM) in multiple antibiotic resistances between group F and group M.

**Data S1**. Original RAs of taxonomy in fresh faeces (F) and manure (M) samples.

**Data S2**. Original RAs and annotation information of sequences with PHI analysis in fresh faeces (F) and manure (M) samples.

**Data S3**. Original RAs of resistance gene type, resistance profile and resistance drug type in fresh faeces (F) and manure (M) samples.

**Data S4**. Annotation information of ARDB analysis in fresh faeces (F) and manure (M) samples.
