# Supplementary figures and images for: Shifts in microbial community, pathogenicity‐related genes and antibiotic resistance genes during dairy manure piled up
Source: Microb Biotechnol. 2020 Mar 23;13(4):1039–53. doi: 10.1111/1751-7915.13551 (PMC7264890; doi:10.1111/1751-7915.13551)

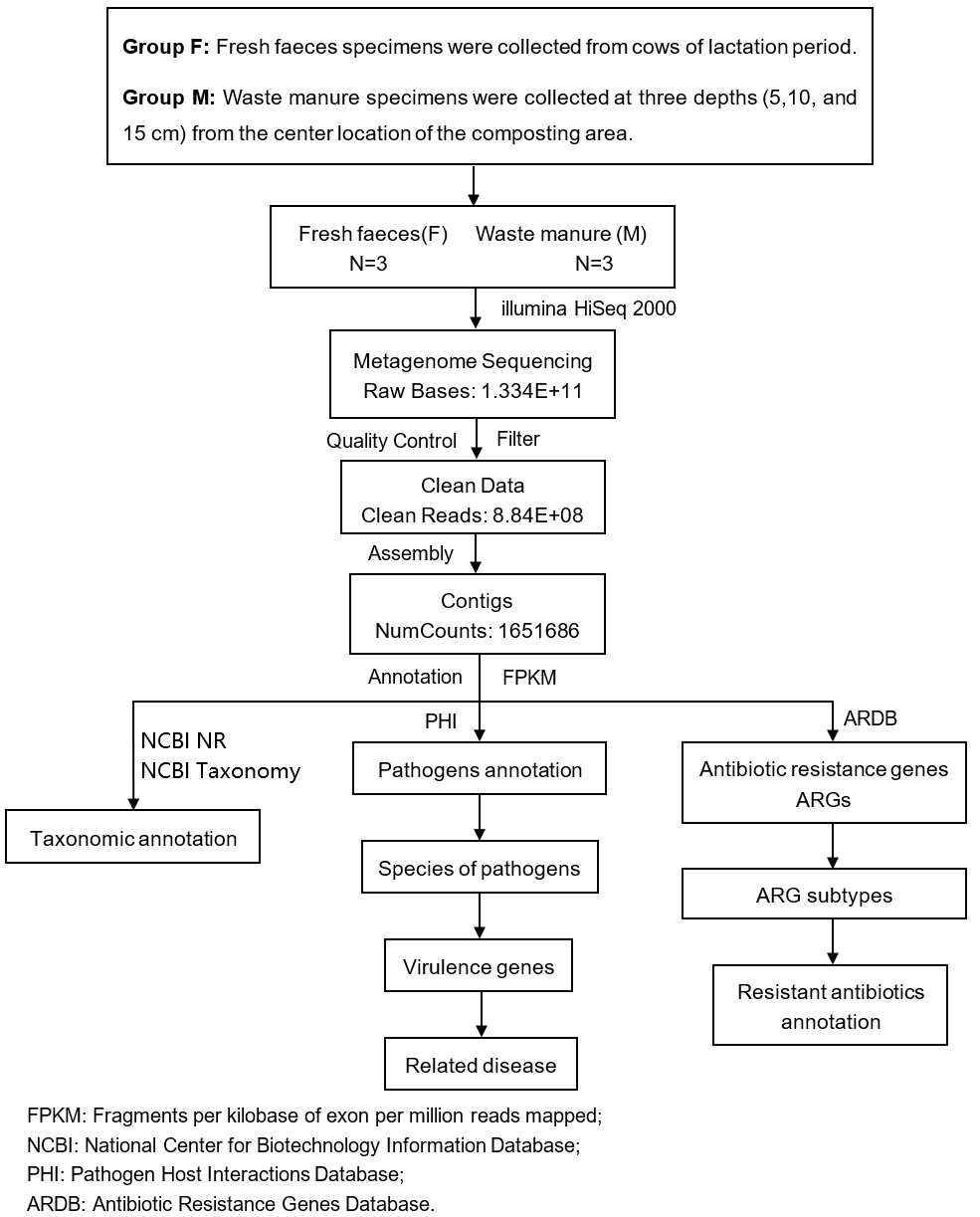

Supplement: Supplementary file 1 — Fig. S1. Flow chart of metagenomic sequencing and analysis at each step. [file MBT2-13-1039-s001.tif]

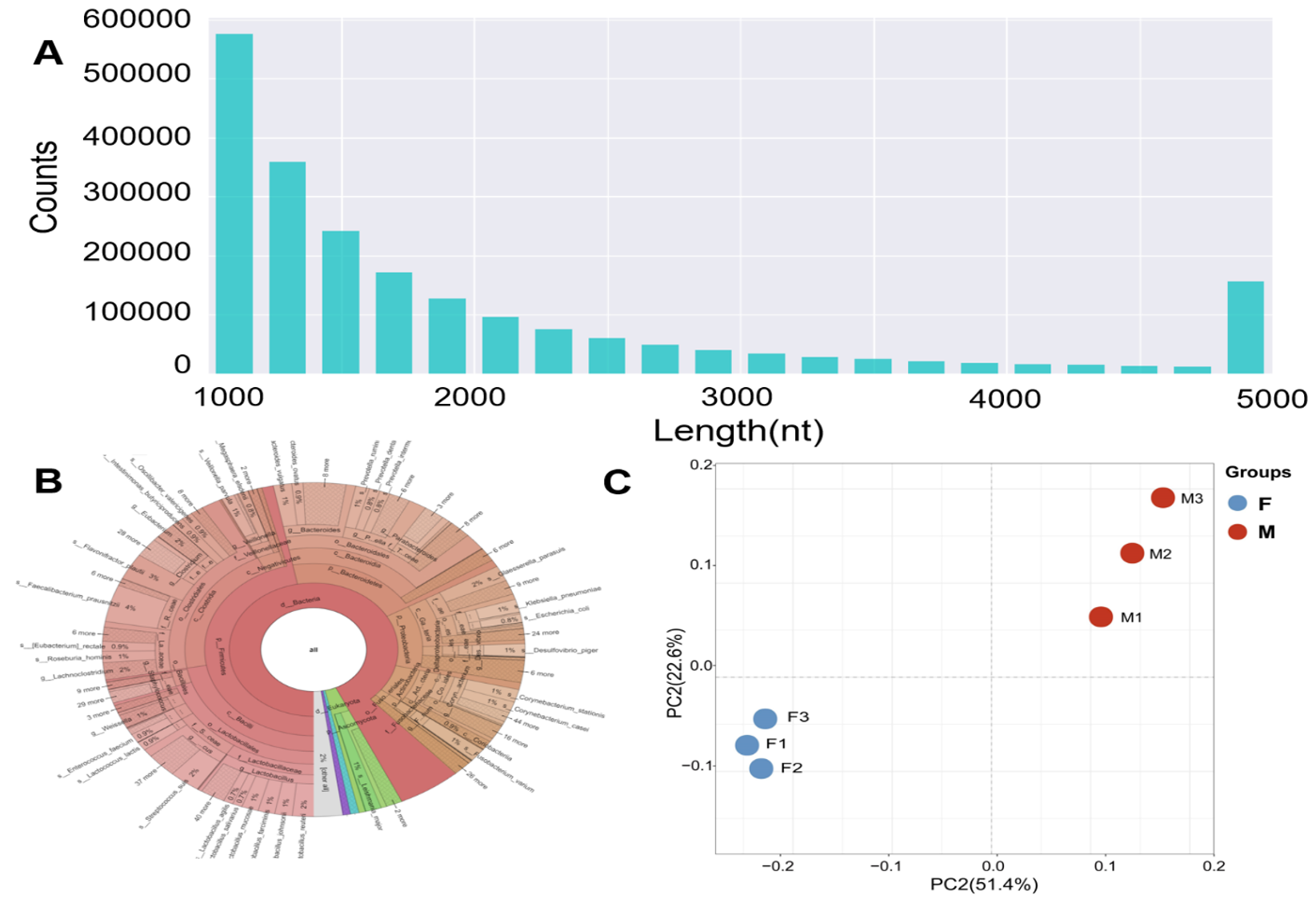

Supplement: Supplementary file 2 — Fig. S2. Quality control and general characteristics of metagenomic sequencing results. (A) Assembly sequence length distribution; (B) species annotation krona frequency distribution; (C) Bray‐Curtis distance of RA data collected on fresh faeces and manure. [file MBT2-13-1039-s002.tif]

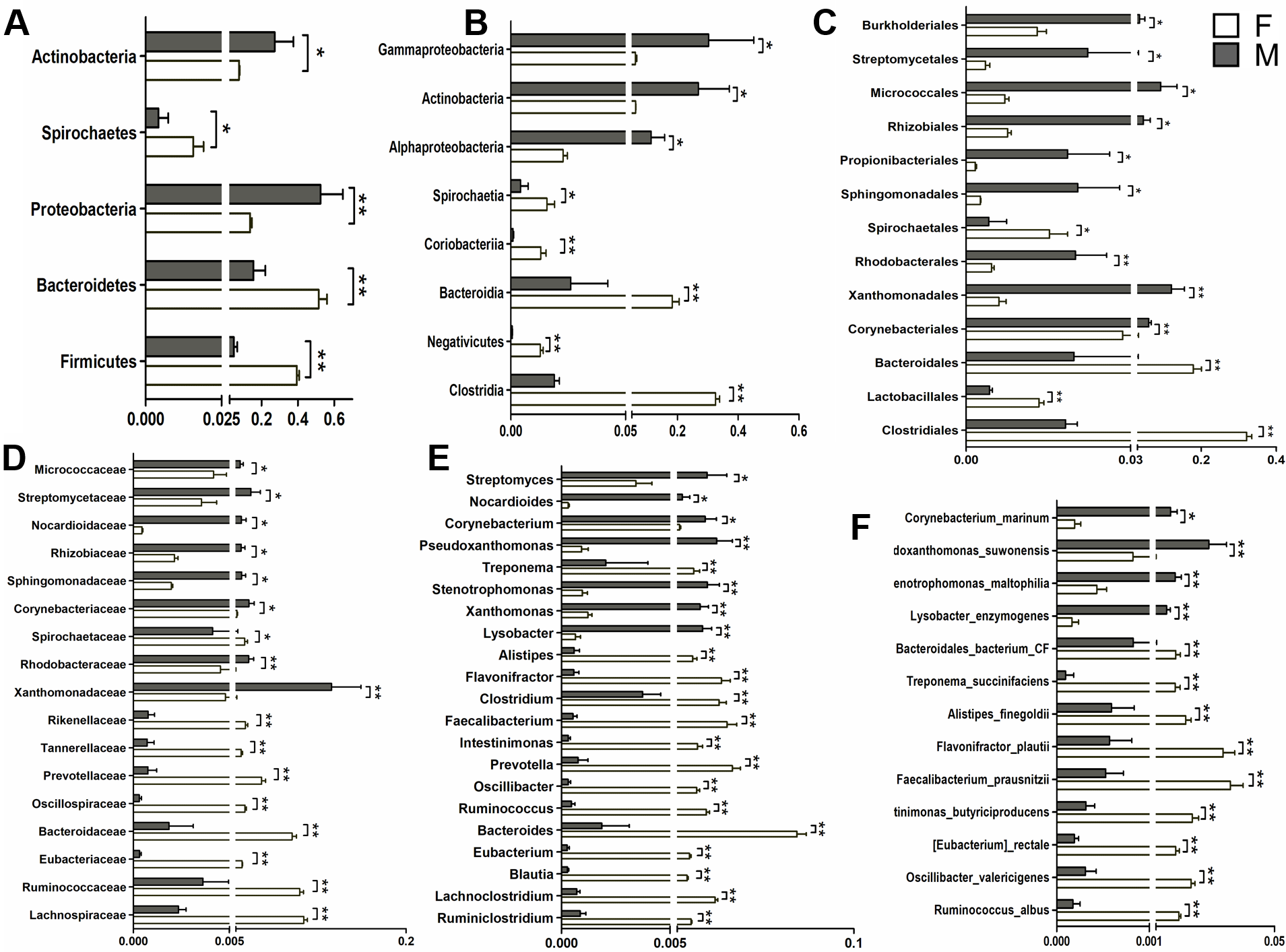

Supplement: Supplementary file 3 — Fig. S3. Column chart analysis of differential RAs of taxonomic annotation between group F and group M. (RAs) ˃1%, *= P < 0.05, **= P < 0.01. (A) Phylum. (B) Class. (C) Order. (D) Family. (E) Genus. (F) Species. [file MBT2-13-1039-s003.tif]

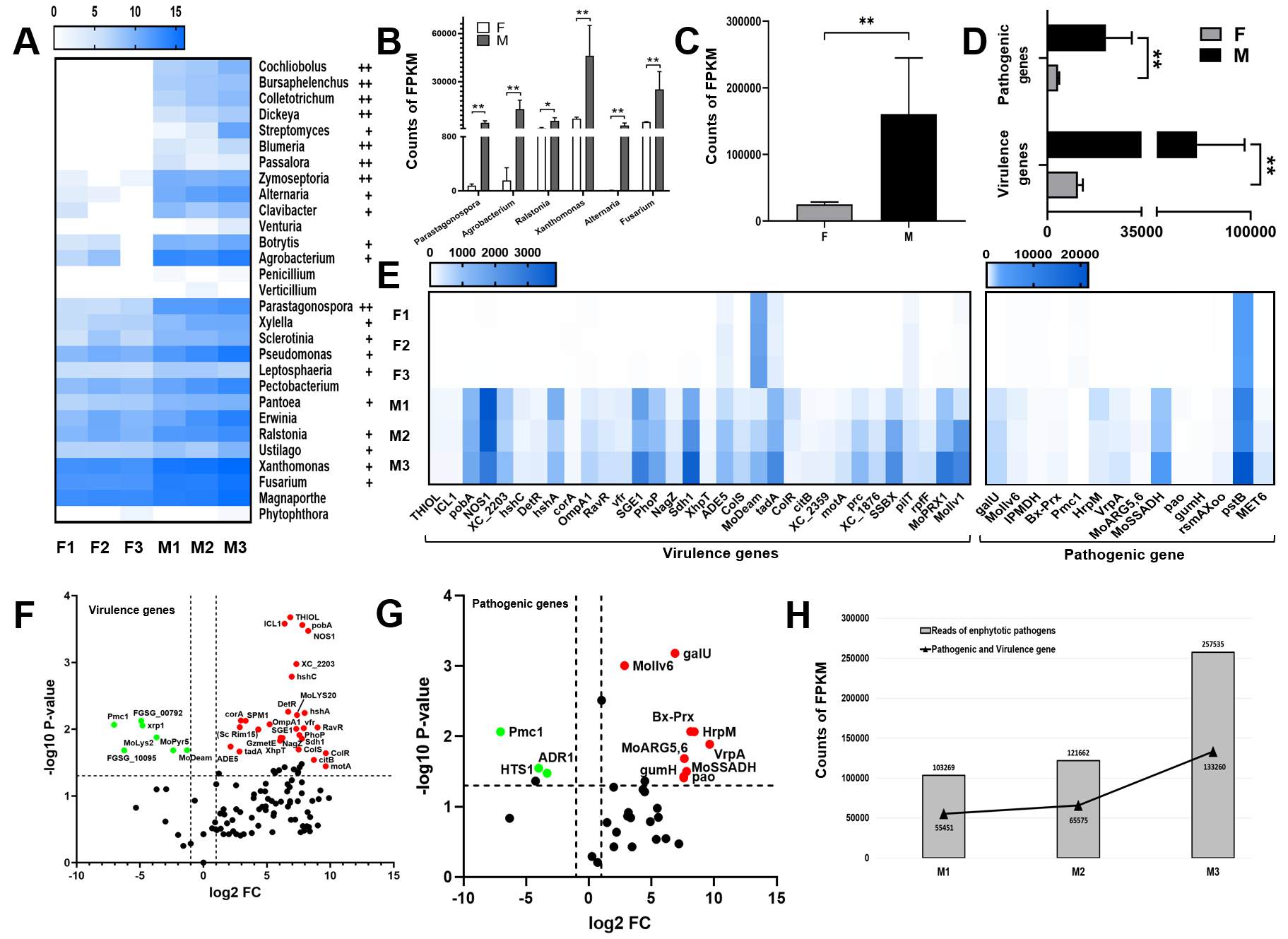

Supplement: Supplementary file 4 — Fig. S4. Comparison of enphytotic genera and virulence gene abundance in fresh faeces and manure samples. (A) Heat map analysis of differential RAs of the enphytotic genus annotation of every sample. A higher abundance is indicated by ‘blue’, and no significant difference is indicated by ‘white’. (B) Column chart analysis of significantly different FPKM of the enphytotic genera between group F and group M. (C) Total FPKM reads of the enphytotic genera between group F and group M. (D&E) Column chart and heat map analysis of differential FPKM of enphytotic virulence genes and pathogenic genes. (F&G) Volcano plot analysis of the differential abundance of enphytotic virulence genes and pathogenic genes between group F and group M. (H) FPKM of enphytotic pathogens, virulence genes and pathogenic genes enhanced in group M with increased manure stacking depth (M1‐5 cm, M2‐10 cm, M3‐15 cm). *,+=P < 0.05, **,++= P < 0.01. [file MBT2-13-1039-s004.tif]
