# Supplementary material for: Shifts in microbial community, pathogenicity‐related genes and antibiotic resistance genes during dairy manure piled up
Source: Microb Biotechnol. 2020 Mar 23;13(4):1039–53. doi: 10.1111/1751-7915.13551 (PMC7264890; doi:10.1111/1751-7915.13551)
Supplement: Supplementary file 5 — Table S1. Data filtering and statistical analysis of metagenomic sequencing. [file MBT2-13-1039-s005.docx]

| **Filter** | | | | | | **Assembly** | | | | | |
| --- | --- | --- | --- | --- | --- | --- | --- | --- | --- | --- | --- |
| **Sample** | **Raw Bases** | **Raw Reads** | **Clean Bases (%)** | **Clean Reads (%)** | **(Max/Mean/Min)** | **NumCounts** | **Min Length (bp)** | **Max Length (bp)** | **Mean Length (bp)** | **Basas** | **Mean GC (%)** |
| F1 | 16000897907 | 106400044 | 15961568465 (99.75%) | 106136816 (99.75%) | 151/150/50 | 210041 | 1000.00 | 283836 | 1989.77 | 4.18E+08 | 44.07 |
| F2 | 18572873170 | 123695764 | 18522470223 (99.73%) | 123356748 (99.73%) | 151/150/50 | 282413 | 1000.00 | 277096 | 2004.65 | 5.66E+08 | 45.39 |
| F3 | 18354115411 | 122150836 | 18306349837 (99.74%) | 121832338 (99.74%) | 151/150/50 | 232495 | 1000.00 | 211121 | 1957.44 | 4.55E+08 | 44.67 |
| M1 | 21625104084 | 143883448 | 21512126254 (99.48%) | 143110190 (99.46%) | 151/150/50 | 250671 | 1000.00 | 355678 | 2772.38 | 6.95E+08 | 58.57 |
| M2 | 22612519130 | 150448764 | 22498454953 (99.50%) | 149670708 (99.48%) | 151/150/50 | 263336 | 1000.00 | 406560 | 2625.96 | 6.92E+08 | 57.94 |
| M3 | 36200729450 | 240692818 | 36086155498 (99.68%) | 239918570 (99.68%) | 151/150/50 | 412730 | 1000.00 | 286228 | 2842.27 | 1.17E+09 | 52.41 |

**Table S1. Data filter and statistics analysis of metagenomic sequencing.**
