# Supplementary material for: Shifts in microbial community, pathogenicity‐related genes and antibiotic resistance genes during dairy manure piled up
Source: Microb Biotechnol. 2020 Mar 23;13(4):1039–53. doi: 10.1111/1751-7915.13551 (PMC7264890; doi:10.1111/1751-7915.13551)
Supplement: Supplementary file 6 — Table S2. The number of microbiome constituents at every taxonomic level between group F and group M. [file MBT2-13-1039-s006.docx]

**Table S2.** **The number of microbiome composition at every taxonomic level between group F and Group M**

|  | Taxonomy | | | | | | |
| --- | --- | --- | --- | --- | --- | --- | --- |
|  | Kingdom | Phylum | Class | Order | Family | Genus | Species |
| Fresh faeces(F) | 4 | 39 | 76 | 166 | 364 | 1071 | 3141 |
| Waste manure(M) | 4 | 39 | 76 | 167 | 369 | 1103 | 3264 |
| Cross | 4 | 39 | 76 | 166 | 362 | 1065 | 3103 |
