# Supplementary material for: Shifts in microbial community, pathogenicity‐related genes and antibiotic resistance genes during dairy manure piled up
Source: Microb Biotechnol. 2020 Mar 23;13(4):1039–53. doi: 10.1111/1751-7915.13551 (PMC7264890; doi:10.1111/1751-7915.13551)
Supplement: Supplementary file 7 — Table S3. The RAs˃1% of microbiome constituents in fresh faeces (F) and manure (M) samples. [file MBT2-13-1039-s007.docx]

**Table S3. The relative abundances(RAs＞1%) of microbiome composition in fresh faeces(F) and waste manure(M) samples.**

| **Taxonomy** |  | | **Relative Abundances(RAs%)** | | | | | |  |
| --- | --- | --- | --- | --- | --- | --- | --- | --- | --- |
|  | **F1** | **F2** | | **F3** | **Mean** | **M1** | **M2** | **M3** | **Mean** |
| **Phylum** |  |  | |  |  |  |  |  |  |
| *Euryarchaeota* | 0.86% | 0.49% | | 1.11% | 0.82% | 2.97% | 1.12% | 0.87% | 1.66% |
| *Spirochaetes* | 1.58% | 1.51% | | 1.02% | 1.37% | 0.06% | 0.63% | 0.51% | 0.40% |
| *Actinobacteria* | 6.45% | 7.18% | | 6.45% | 6.70% | 40.43% | 17.13% | 23.86% | 27.14% |
| *Proteobacteria* | 10.96% | 13.08% | | 11.82% | 11.96% | 40.32% | 57.67% | 54.73% | 50.91% |
| *Firmicutes* | 33.12% | 36.51% | | 33.50% | 34.37% | 7.34% | 3.55% | 3.70% | 4.86% |
| *Bacteroidetes* | 47.02% | 41.23% | | 46.08% | 44.78% | 8.88% | 19.89% | 16.32% | 15.03% |
| **Class** |  |  | |  |  |  |  |  |  |
| *Methanobacteria* | 1.01% | 0.49% | | 1.44% | 0.98% | 1.53% | 0.07% | 0.43% | 0.68% |
| *Cytophagia* | 1.04% | 0.84% | | 1.03% | 0.97% | 1.47% | 0.71% | 0.79% | 0.99% |
| *Coriobacteriia* | 1.37% | 1.97% | | 1.57% | 1.64% | 0.15% | 0.12% | 0.08% | 0.11% |
| *Negativicutes* | 1.44% | 1.78% | | 1.60% | 1.61% | 0.06% | 0.08% | 0.07% | 0.07% |
| *Deltaproteobacteria* | 1.81% | 2.12% | | 1.89% | 1.94% | 1.90% | 5.55% | 1.22% | 2.89% |
| *Betaproteobacteria* | 1.99% | 2.53% | | 2.12% | 2.21% | 5.66% | 8.54% | 3.16% | 5.79% |
| *Spirochaetia* | 2.31% | 2.14% | | 1.49% | 1.98% | 0.07% | 0.79% | 0.61% | 0.49% |
| *Flavobacteriia* | 2.42% | 1.74% | | 2.23% | 2.13% | 1.83% | 1.59% | 2.21% | 1.87% |
| *Alphaproteobacteria* | 2.66% | 3.15% | | 2.78% | 2.86% | 17.09% | 15.49% | 7.09% | 13.22% |
| *Bacilli* | 4.52% | 4.11% | | 4.10% | 4.24% | 5.19% | 1.32% | 1.77% | 2.76% |
| *Gammaproteobacteria* | 7.07% | 8.11% | | 8.04% | 7.74% | 17.08% | 37.56% | 49.50% | 34.72% |
| *Actinobacteria* | 7.85% | 7.98% | | 7.61% | 7.81% | 44.83% | 20.97% | 27.81% | 31.20% |
| *Bacteroidia* | 25.16% | 19.98% | | 23.86% | 23.00% | 1.02% | 4.71% | 3.31% | 3.01% |
| *Clostridia* | 39.37% | 43.07% | | 40.25% | 40.90% | 2.13% | 2.51% | 1.94% | 2.19% |
| **Order** |  |  | |  |  |  |  |  |  |
| *Myxococcales* | 0.29% | 0.36% | | 0.31% | 0.32% | 1.60% | 4.29% | 0.38% | 2.09% |
| *Lactobacillales* | 2.08% | 1.89% | | 1.88% | 1.95% | 0.69% | 0.56% | 0.66% | 0.63% |
| *Spirochaetales* | 2.57% | 2.46% | | 1.68% | 2.24% | 0.08% | 0.99% | 0.73% | 0.60% |
| *Cytophagales* | 1.20% | 0.99% | | 1.21% | 1.13% | 1.97% | 0.90% | 0.97% | 1.28% |
| *Bacillales* | 2.87% | 2.74% | | 2.71% | 2.77% | 6.21% | 1.07% | 1.44% | 2.91% |
| *Sphingomonadales* | 0.37% | 0.40% | | 0.38% | 0.39% | 4.26% | 3.30% | 1.65% | 3.07% |
| *Rhodobacterales* | 0.61% | 0.77% | | 0.66% | 0.68% | 3.50% | 3.60% | 1.87% | 2.99% |
| *Enterobacterales* | 1.42% | 1.81% | | 1.61% | 1.61% | 0.85% | 1.56% | 2.22% | 1.54% |
| *Clostridiales* | 44.69% | 50.15% | | 46.69% | 47.18% | 2.77% | 3.05% | 2.26% | 2.69% |
| *Propionibacteriales* | 0.21% | 0.28% | | 0.25% | 0.25% | 4.24% | 1.74% | 2.36% | 2.78% |
| *Streptomycetales* | 0.41% | 0.65% | | 0.51% | 0.52% | 5.45% | 2.01% | 2.57% | 3.34% |
| *Flavobacteriales* | 2.79% | 2.06% | | 2.62% | 2.49% | 2.45% | 2.02% | 2.71% | 2.39% |
| *Burkholderiales* | 1.68% | 2.21% | | 1.84% | 1.91% | 5.82% | 7.33% | 2.95% | 5.37% |
| *Bacteroidales* | 28.31% | 23.05% | | 27.34% | 26.24% | 0.83% | 4.46% | 3.28% | 2.86% |
| *Rhizobiales* | 1.01% | 1.24% | | 1.09% | 1.11% | 8.55% | 8.75% | 3.46% | 6.92% |
| *Alteromonadales* | 0.62% | 0.72% | | 0.83% | 0.72% | 0.49% | 7.97% | 5.38% | 4.61% |
| *Corynebacteriales* | 4.89% | 3.96% | | 3.73% | 4.19% | 10.73% | 8.37% | 7.81% | 8.97% |
| *Oceanospirillales* | 0.35% | 0.46% | | 0.60% | 0.47% | 0.63% | 6.05% | 8.12% | 4.93% |
| *Micrococcales* | 0.91% | 1.15% | | 1.07% | 1.04% | 21.75% | 7.64% | 12.10% | 13.83% |
| *Pseudomonadales* | 1.88% | 1.94% | | 1.92% | 1.91% | 3.58% | 6.35% | 15.66% | 8.53% |
| *Xanthomonadales* | 0.86% | 0.71% | | 1.07% | 0.88% | 13.57% | 17.99% | 21.44% | 17.67% |
| **Family** |  |  | |  |  |  |  |  |  |
| *Flavobacteriaceae* | 4.12% | 3.08% | | 3.86% | 3.69% | 3.35% | 2.73% | 3.73% | 3.27% |
| *Porphyromonadaceae* | 1.91% | 1.52% | | 1.88% | 1.77% | 0.34% | 2.84% | 2.34% | 1.84% |
| *Methanobacteriaceae* | 1.80% | 0.92% | | 2.61% | 1.78% | 2.88% | 0.13% | 0.76% | 1.26% |
| *Planococcaceae* | 0.60% | 0.52% | | 0.51% | 0.54% | 7.33% | 0.53% | 0.74% | 2.87% |
| *Polyangiaceae* | 0.08% | 0.08% | | 0.08% | 0.08% | 0.42% | 4.76% | 0.11% | 1.76% |
| *Mycobacteriaceae* | 5.92% | 4.06% | | 3.84% | 4.61% | 8.09% | 6.04% | 3.78% | 5.97% |
| *Caulobacteraceae* | 0.14% | 0.18% | | 0.16% | 0.16% | 4.68% | 1.33% | 0.40% | 2.14% |
| *Alteromonadaceae* | 0.32% | 0.43% | | 0.58% | 0.45% | 0.41% | 10.13% | 4.42% | 4.99% |
| *Comamonadaceae* | 0.84% | 1.20% | | 0.94% | 0.99% | 1.86% | 3.16% | 1.05% | 2.02% |
| *Halomonadaceae* | 0.25% | 0.34% | | 0.59% | 0.40% | 0.63% | 7.99% | 10.64% | 6.42% |
| *Pseudomonadaceae* | 2.43% | 2.50% | | 2.40% | 2.44% | 4.84% | 7.77% | 15.39% | 9.33% |
| *Intrasporangiaceae* | 0.11% | 0.07% | | 0.14% | 0.11% | 16.08% | 3.90% | 6.38% | 8.79% |
| *Micrococcaceae* | 0.79% | 1.15% | | 0.93% | 0.96% | 3.02% | 1.40% | 1.96% | 2.13% |
| *Streptomycetaceae* | 0.63% | 1.03% | | 0.78% | 0.82% | 7.69% | 2.86% | 3.65% | 4.74% |
| *Nocardioidaceae* | 0.09% | 0.11% | | 0.11% | 0.10% | 3.82% | 1.31% | 2.05% | 2.39% |
| *Rhizobiaceae* | 0.44% | 0.55% | | 0.48% | 0.49% | 3.37% | 2.44% | 1.23% | 2.35% |
| *Sphingomonadaceae* | 0.43% | 0.49% | | 0.44% | 0.45% | 3.50% | 2.64% | 1.36% | 2.50% |
| *Corynebacteriaceae* | 1.22% | 1.62% | | 1.44% | 1.43% | 3.11% | 4.20% | 5.13% | 4.15% |
| *Spirochaetaceae* | 3.93% | 3.85% | | 2.56% | 3.45% | 0.11% | 1.40% | 1.03% | 0.85% |
| *Rhodobacteraceae* | 0.92% | 1.19% | | 1.00% | 1.04% | 4.89% | 5.07% | 2.62% | 4.19% |
| *Xanthomonadaceae* | 1.08% | 0.82% | | 1.38% | 1.09% | 18.10% | 24.18% | 29.11% | 23.80% |
| *Rikenellaceae* | 3.97% | 3.02% | | 3.87% | 3.62% | 0.09% | 0.23% | 0.16% | 0.16% |
| *Tannerellaceae* | 2.77% | 2.25% | | 2.64% | 2.56% | 0.07% | 0.22% | 0.15% | 0.15% |
| *Prevotellaceae* | 8.46% | 6.98% | | 8.25% | 7.90% | 0.06% | 0.25% | 0.17% | 0.16% |
| *Oscillospiraceae* | 3.18% | 4.07% | | 3.43% | 3.56% | 0.04% | 0.09% | 0.07% | 0.06% |
| *Bacteroidaceae* | 16.44% | 15.03% | | 16.10% | 15.86% | 0.12% | 0.65% | 0.39% | 0.39% |
| *Eubacteriaceae* | 2.56% | 3.04% | | 2.72% | 2.77% | 0.06% | 0.09% | 0.06% | 0.07% |
| *Ruminococcaceae* | 17.10% | 19.62% | | 17.12% | 17.95% | 0.58% | 1.08% | 0.62% | 0.76% |
| *Lachnospiraceae* | 17.45% | 20.27% | | 19.12% | 18.95% | 0.43% | 0.58% | 0.47% | 0.49% |
| **Genus** |  |  | |  |  |  |  |  |  |
| *Clostridium* | 7.60% | 6.05% | | 7.75% | 7.14% | 1.56% | 1.44% | 1.12% | 1.37% |
| *Nocardioides* | 0.06% | 0.09% | | 0.09% | 0.08% | 3.95% | 1.86% | 2.51% | 2.77% |
| *Methanobrevibacter* | 2.03% | 1.00% | | 2.95% | 1.99% | 4.16% | 0.25% | 1.26% | 1.89% |
| *Xanthomonas* | 0.30% | 0.28% | | 0.37% | 0.32% | 4.18% | 7.92% | 7.27% | 6.46% |
| *Lysobacter* | 0.17% | 0.11% | | 0.23% | 0.17% | 4.61% | 8.22% | 7.96% | 6.93% |
| *Corynebacterium* | 1.40% | 1.81% | | 1.66% | 1.62% | 4.64% | 8.96% | 8.74% | 7.45% |
| *Stenotrophomonas* | 0.25% | 0.19% | | 0.31% | 0.25% | 4.94% | 9.60% | 9.22% | 7.92% |
| *Brevundimonas* | 0.05% | 0.06% | | 0.05% | 0.05% | 5.29% | 1.35% | 0.34% | 2.33% |
| *Pseudoxanthomonas* | 0.24% | 0.16% | | 0.32% | 0.24% | 5.87% | 11.94% | 11.54% | 9.78% |
| *Pseudomonas* | 2.69% | 2.65% | | 2.62% | 2.65% | 7.01% | 15.85% | 24.77% | 15.87% |
| *Planococcus* | 0.34% | 0.32% | | 0.32% | 0.33% | 10.66% | 1.02% | 1.08% | 4.25% |
| *Streptomyces* | 0.70% | 1.11% | | 0.87% | 0.89% | 10.94% | 5.83% | 5.91% | 7.56% |
| *Mycobacterium* | 6.76% | 4.54% | | 4.41% | 5.24% | 12.02% | 12.88% | 6.43% | 10.44% |
| *Serinicoccus* | 0.08% | 0.04% | | 0.11% | 0.08% | 18.71% | 6.19% | 8.72% | 11.20% |
| *Alistipes* | 3.81% | 2.84% | | 3.73% | 3.46% | 0.11% | 0.37% | 0.20% | 0.23% |
| *Blautia* | 2.53% | 2.92% | | 2.62% | 2.69% | 0.07% | 0.15% | 0.09% | 0.11% |
| *Eubacterium* | 2.78% | 3.28% | | 3.01% | 3.02% | 0.07% | 0.17% | 0.08% | 0.10% |
| *Ruminiclostridium* | 3.26% | 3.34% | | 3.14% | 3.25% | 0.21% | 0.52% | 0.27% | 0.33% |
| *Treponema* | 4.19% | 3.96% | | 2.66% | 3.60% | 0.10% | 1.86% | 0.56% | 0.84% |
| *Oscillibacter* | 3.63% | 4.54% | | 3.94% | 4.04% | 0.06% | 0.18% | 0.11% | 0.12% |
| *Intestinimonas* | 3.51% | 4.97% | | 4.00% | 4.16% | 0.07% | 0.19% | 0.10% | 0.12% |
| *Ruminococcus* | 5.78% | 5.18% | | 5.21% | 5.39% | 0.09% | 0.27% | 0.17% | 0.18% |
| *Lachnoclostridium* | 6.09% | 7.07% | | 6.62% | 6.59% | 0.18% | 0.38% | 0.25% | 0.27% |
| *Prevotella* | 9.67% | 7.80% | | 9.48% | 8.98% | 0.09% | 0.53% | 0.28% | 0.30% |
| *Flavonifractor* | 6.34% | 8.98% | | 7.14% | 7.48% | 0.11% | 0.37% | 0.19% | 0.22% |
| *Faecalibacterium* | 6.97% | 9.90% | | 7.93% | 8.27% | 0.11% | 0.32% | 0.18% | 0.20% |
| *Bacteroides* | 18.79% | 16.81% | | 18.48% | 18.02% | 0.18% | 1.38% | 0.66% | 0.74% |
| **Species** |  |  | |  |  |  |  |  |  |
| *Treponema_succinifaciens* | 7.40% | 6.41% | | 4.53% | 6.11% | 0.02% | 0.18% | 0.06% | 0.08% |
| *Bacteroidales_bacterium_CF* | 7.75% | 4.40% | | 6.80% | 6.32% | 0.38% | 1.20% | 0.58% | 0.72% |
| *Ruminococcus_albus* | 8.29% | 6.36% | | 6.94% | 7.20% | 0.09% | 0.21% | 0.15% | 0.15% |
| *[Eubacterium]_rectale* | 5.41% | 6.26% | | 7.05% | 6.24% | 0.15% | 0.22% | 0.13% | 0.17% |
| *Alistipes_finegoldii* | 10.83% | 6.82% | | 9.97% | 9.21% | 0.36% | 0.77% | 0.42% | 0.52% |
| *Oscillibacter_valericigenes* | 10.32% | 10.91% | | 10.54% | 10.59% | 0.19% | 0.38% | 0.24% | 0.27% |
| *Intestinimonas_butyriciproducens* | 9.98% | 11.94% | | 10.70% | 10.87% | 0.23% | 0.39% | 0.22% | 0.28% |
| *Flavonifractor_plautii* | 18.03% | 21.56% | | 19.10% | 19.56% | 0.35% | 0.76% | 0.39% | 0.50% |
| *Faecalibacterium_prausnitzii* | 19.83% | 23.77% | | 21.23% | 21.61% | 0.35% | 0.66% | 0.38% | 0.46% |
| *Marinobacter_hydrocarbonoclasticus* | 0.11% | 0.13% | | 0.36% | 0.20% | 0.32% | 13.06% | 3.83% | 5.74% |
| *Marinobacter_sp._LQ44* | 0.14% | 0.15% | | 0.43% | 0.24% | 0.42% | 21.68% | 4.72% | 8.94% |
| *Lysobacter_enzymogenes* | 0.13% | 0.05% | | 0.16% | 0.11% | 4.21% | 5.00% | 4.86% | 4.69% |
| *Corynebacterium_marinum* | 0.13% | 0.09% | | 0.19% | 0.13% | 5.50% | 5.50% | 7.10% | 6.03% |
| *Stenotrophomonas_maltophilia* | 0.32% | 0.21% | | 0.37% | 0.30% | 6.84% | 8.22% | 8.09% | 7.72% |
| *Halomonas_campaniensis* | 0.03% | 0.03% | | 0.05% | 0.04% | 0.09% | 0.80% | 9.86% | 3.58% |
| *Serinicoccus_sp._JLT9* | 0.23% | 0.10% | | 0.30% | 0.21% | 59.21% | 12.84% | 18.24% | 30.10% |
| *Pseudomonas_stutzeri* | 0.46% | 0.47% | | 0.54% | 0.49% | 5.03% | 6.49% | 19.72% | 10.41% |
| *Pseudoxanthomonas_suwonensis* | 0.61% | 0.35% | | 0.75% | 0.57% | 16.25% | 21.60% | 21.02% | 19.62% |
