# Supplementary material for: Shifts in microbial community, pathogenicity‐related genes and antibiotic resistance genes during dairy manure piled up
Source: Microb Biotechnol. 2020 Mar 23;13(4):1039–53. doi: 10.1111/1751-7915.13551 (PMC7264890; doi:10.1111/1751-7915.13551)
Supplement: Supplementary file 8 — Table S4. Statistical analysis of the difference in taxonomic composition between group F and group M. [file MBT2-13-1039-s008.docx]

**Table S4.** **Statistical analysis the difference of taxonomic composition between** **Group F and Group M.**

|  |  | | |
| --- | --- | --- | --- |
|  | **Name** | **P-value** | **Fold change** |
| **Phylum** | *Actinobacteria* | 0.03 | 3.56 |
|  | *Proteobacteria* | 0.006 | 3.83 |
|  | *Firmicutes* | 1.16E-05 | 0.12 |
|  | *Bacteroidetes* | 0.001 | 0.3 |
|  | *Spirochaetes* | 0.001 | 0.27 |
| **Class** | *Alphaproteobacteria* | 0.02 | 4.99 |
|  | *Actinobacteria* | 0.02 | 4.33 |
|  | *Gammaproteobacteria* | 0.04 | 4.91 |
|  | *Clostridia* | 2.66E-06 | 0.06 |
|  | *Negativicutes* | 8.91E-05 | 0.05 |
|  | *Bacteroidia* | 0.000601 | 0.14 |
|  | *Spirochaetia* | 0.012896 | 0.27 |
| **Order** | *Corynebacteriales* | 0.002 | 2.11 |
|  | *Xanthomonadales* | 0.005 | 20.15 |
|  | *Rhodobacterales* | 0.009 | 4.3 |
|  | *Sphingomonadales* | 0.016 | 7.76 |
|  | *Propionibacteriales* | 0.019 | 11.07 |
|  | *Micrococcales* | 0.03 | 12.99 |
|  | *Streptomycetales* | 0.04 | 6.26 |
|  | *Burkholderiales* | 0.048 | 2.76 |
|  | *Rhizobiales* | 0.02 | 6.09 |
|  | *Clostridiales* | 2.84E-06 | 0.06 |
|  | *Lactobacillales* | 0.0001 | 0.32 |
|  | *Bacteroidales* | 0.0004 | 0.11 |
|  | *Spirochaetales* | 0.014 | 0.27 |
| **Family** | *Xanthomonadaceae* | 0.005 | 23.91 |
|  | *Rhodobacteraceae* | 0.009 | 4.36 |
|  | *Corynebacteriaceae* | 0.018 | 3.21 |
|  | *Sphingomonadaceae* | 0.019 | 5.93 |
|  | *Rhizobiaceae* | 0.026 | 5.12 |
|  | *Nocardioidaceae* | 0.027 | 24.97 |
|  | *Streptomycetaceae* | 0.042 | 6.26 |
|  | *Micrococcaceae* | 0.045 | 2.41 |
|  | *Lachnospiraceae* | 5.55E-06 | 0.03 |
|  | *Ruminococcaceae* | 7.76E-06 | 0.05 |
|  | *Eubacteriaceae* | 1.05E-05 | 0.03 |
|  | *Bacteroidaceae* | 2.33E-05 | 0.03 |
|  | *Oscillospiraceae* | 7.36E-05 | 0.02 |
|  | *Prevotellaceae* | 0.0001 | 0.02 |
|  | *Tannerellaceae* | 0.0003 | 0.06 |
|  | *Rikenellaceae* | 0.0006 | 0.05 |
|  | *Spirochaetaceae* | 0.015 | 0.27 |
| **Genus** | *Lysobacter* | 0.003 | 28.67 |
|  | *Pseudoxanthomonas* | 0.007 | 28.08 |
|  | *Nocardioides* | 0.028 | 25.43 |
|  | *Stenotrophomonas* | 0.005 | 21.97 |
|  | *Xanthomonas* | 0.004 | 14.17 |
|  | *Streptomyces* | 0.043 | 6.18 |
|  | *Corynebacterium* | 0.018 | 3.21 |
|  | *Treponema* | 0.005 | 0.15 |
|  | *Clostridium* | 0.001 | 0.14 |
|  | *Ruminiclostridium* | 1.37E-06 | 0.07 |
|  | *Alistipes* | 0.0006 | 0.04 |
|  | *Lachnoclostridium* | 9.76E-06 | 0.03 |
|  | *Blautia* | 1.01E-05 | 0.03 |
|  | *Bacteroides* | 2.33E-05 | 0.03 |
|  | *Eubacterium* | 1.65E-05 | 0.02 |
|  | *Prevotella* | 0.0002 | 0.02 |
|  | *Ruminococcus* | 2.43E-05 | 0.02 |
|  | *Oscillibacter* | 7.36E-05 | 0.02 |
|  | *Flavonifractor* | 0.0005 | 0.02 |
|  | *Intestinimonas* | 0.0005 | 0.02 |
|  | *Faecalibacterium* | 0.0005 | 0.02 |
| **Species** | *Lysobacter_enzymogenes* | 0.003 | 33.23 |
|  | *Stenotrophomonas_maltophilia* | 0.004 | 20.68 |
|  | *Pseudoxanthomonas_suwonensis* | 0.006 | 27.77 |
|  | *Corynebacterium_marinum* | 0.01 | 36.43 |
|  | *Ruminococcus_albus* | 2.33E-05 | 0.02 |
|  | *Oscillibacter_valericigenes* | 7.36E-05 | 0.02 |
|  | *[Eubacterium]_rectale* | 0.0005 | 0.02 |
|  | *Intestinimonas_butyriciproducens* | 0.0005 | 0.02 |
|  | *Faecalibacterium_prausnitzii* | 0.0005 | 0.02 |
|  | *Flavonifractor_plautii* | 0.0005 | 0.02 |
|  | *Alistipes_finegoldii* | 0.0006 | 0.04 |
|  | *Treponema_succinifaciens* | 0.002 | 0.01 |
|  | *Bacteroidales_bacterium_CF* | 0.002 | 0.09 |

**Addation: RA＞1%, P＜0.05, Fold change＞1 means up-regulation,**

**Fold change＜1 means down-regulation.**
