# Supplementary material for: Shifts in microbial community, pathogenicity‐related genes and antibiotic resistance genes during dairy manure piled up
Source: Microb Biotechnol. 2020 Mar 23;13(4):1039–53. doi: 10.1111/1751-7915.13551 (PMC7264890; doi:10.1111/1751-7915.13551)
Supplement: Supplementary file 9 — Table S5. Differential number of contigs (FPKM) in ARGs and ARG subtypes between group F and group M. [file MBT2-13-1039-s009.docx]

**Table S5.** **Differential number of contigs(FPKM) in antibiotic resistance genes(ARGs) and ARG subtypes between Group F and Group M.**

| **Antibiotic resistance genes(ARGs)** | **Number of contigs(FPKM)** | | | | | | **Fold Change** | **P-value** |
| --- | --- | --- | --- | --- | --- | --- | --- | --- |
|  | **F1** | **F2** | **F3** | **M1** | **M2** | **M3** |  |  |
| lsa | 7 | 7 | 6 | 0 | 0 | 0 | 0.05 | 0.000 |
| vang | 739 | 941 | 736 | 38 | 53 | 136 | 0.09 | 0.001 |
| mexxy | 20 | 14 | 23 | 0 | 0 | 2 | 0.04 | 0.003 |
| rosab | 1 | 0 | 1 | 46 | 52 | 76 | 87.00 | 0.003 |
| tet_flavo | 465 | 545 | 567 | 252 | 67 | 149 | 0.30 | 0.004 |
| vana | 4 | 2 | 3 | 0 | 0 | 0 | 0.11 | 0.007 |
| smedef | 1 | 2 | 1 | 449 | 234 | 281 | 241.00 | 0.008 |
| aac | 16 | 6 | 4 | 53 | 48 | 83 | 7.08 | 0.010 |
| vat | 392 | 322 | 313 | 819 | 543 | 679 | 1.99 | 0.016 |
| mls_abc | 525 | 834 | 604 | 2375 | 2240 | 4341 | 4.56 | 0.027 |
| aph | 1 | 0 | 0 | 39 | 129 | 117 | 285.00 | 0.028 |
| cml | 0 | 0 | 0 | 16 | 7 | 6 | 29.00 | 0.038 |
| bla_a | 0 | 0 | 0 | 34 | 10 | 44 | 88.00 | 0.044 |
| vand | 7 | 32 | 22 | 0 | 1 | 0 | 0.02 | 0.051 |
| ant | 0 | 1 | 1 | 281 | 50 | 258 | 294.50 | 0.056 |
| tet_efflux | 0 | 0 | 0 | 43 | 12 | 14 | 69.00 | 0.083 |
| bcr | 0 | 0 | 0 | 8 | 5 | 23 | 36.00 | 0.097 |
| vanc | 11 | 20 | 7 | 0 | 8 | 0 | 0.21 | 0.099 |
| ceo | 0 | 0 | 0 | 3 | 3 | 11 | 17.00 | 0.101 |
| ykk | 0 | 0 | 0 | 91 | 10 | 48 | 149.00 | 0.101 |
| ere | 0 | 0 | 0 | 1 | 22 | 15 | 38.00 | 0.109 |
| erm | 0 | 0 | 2 | 49 | 16 | 123 | 94.00 | 0.122 |
| vanb | 0 | 3 | 2 | 0 | 0 | 0 | 0.20 | 0.132 |
| dfra | 0 | 3 | 3 | 16 | 136 | 354 | 84.33 | 0.167 |
| tet_rpp | 69 | 202 | 118 | 97 | 701 | 1220 | 5.19 | 0.172 |
| mls_hdr | 0 | 0 | 0 | 6 | 1 | 18 | 25.00 | 0.174 |
| mexvw | 0 | 0 | 0 | 34 | 24 | 182 | 240.00 | 0.192 |
| tet_xprt | 0 | 0 | 1 | 3 | 8 | 35 | 46.00 | 0.206 |
| lnu | 0 | 0 | 0 | 1 | 6 | 28 | 35.00 | 0.232 |
| bla_c | 1 | 0 | 3 | 1 | 5 | 13 | 4.75 | 0.241 |
| mexcd | 1 | 0 | 0 | 2 | 3 | 23 | 28.00 | 0.259 |
| mls_mfs | 0 | 0 | 0 | 1 | 11 | 69 | 81.00 | 0.272 |
| mdtef | 0 | 0 | 0 | 1 | 6 | 0 | 7.00 | 0.277 |
| mexab | 0 | 0 | 0 | 0 | 4 | 28 | 32.00 | 0.289 |
| smeabc | 0 | 0 | 0 | 7 | 0 | 1 | 8.00 | 0.289 |
| bla_d | 0 | 0 | 0 | 6 | 6 | 141 | 153.00 | 0.320 |
| adeabc | 0 | 0 | 1 | 0 | 18 | 213 | 231.00 | 0.324 |
| mexef | 0 | 0 | 1 | 4 | 123 | 6 | 133.00 | 0.326 |
| qac | 0 | 0 | 0 | 0 | 0 | 15 | 15.00 | 0.374 |
| mph | 0 | 0 | 0 | 7 | 0 | 0 | 7.00 | 0.374 |
| fos | 0 | 0 | 0 | 0 | 0 | 3 | 3.00 | 0.374 |
| arna | 0 | 1 | 2 | 0 | 0 | 1 | 0.33 | 0.374 |
| vane | 19 | 31 | 15 | 24 | 19 | 33 | 1.17 | 0.593 |
| tet_mod | 0 | 0 | 1 | 0 | 0 | 2 | 2.00 | 0.678 |
| catb | 8 | 1 | 8 | 2 | 1 | 14 | 1.00 | 1.000 |
| **Antibiotic resistance gene subtypes(ARG subtypes)** | | | | | | | | |
| lsa | 7 | 7 | 6 | 0 | 0 | 0 | 0.05 | 0.000 |
| vanrg | 40 | 42 | 49 | 1 | 2 | 2 | 0.04 | 0.000 |
| aph33ia | 1 | 0 | 0 | 23 | 28 | 23 | 37.00 | 0.000 |
| vanug | 675 | 827 | 671 | 34 | 49 | 134 | 0.10 | 0.000 |
| vatb | 141 | 157 | 158 | 495 | 427 | 536 | 3.19 | 0.000 |
| vantg | 6 | 6 | 4 | 0 | 0 | 0 | 0.06 | 0.001 |
| mexy | 20 | 14 | 23 | 0 | 0 | 2 | 0.03 | 0.003 |
| rosa | 1 | 0 | 1 | 46 | 52 | 76 | 58.00 | 0.003 |
| tet37 | 465 | 545 | 567 | 252 | 67 | 149 | 0.30 | 0.004 |
| aac2ib | 0 | 0 | 0 | 9 | 8 | 14 | 31.00 | 0.005 |
| smef | 1 | 0 | 1 | 45 | 28 | 54 | 42.33 | 0.005 |
| vana | 4 | 2 | 3 | 0 | 0 | 0 | 0.10 | 0.007 |
| tlrc | 39 | 156 | 85 | 898 | 742 | 1495 | 11.16 | 0.015 |
| smed | 0 | 1 | 0 | 291 | 190 | 120 | 300.50 | 0.016 |
| tetw | 50 | 109 | 65 | 1 | 1 | 14 | 0.07 | 0.019 |
| cara | 124 | 152 | 99 | 460 | 564 | 937 | 5.22 | 0.022 |
| vang | 18 | 28 | 10 | 0 | 0 | 0 | 0.02 | 0.023 |
| aac6ic | 0 | 0 | 0 | 10 | 17 | 6 | 33.00 | 0.027 |
| aac3iib | 0 | 0 | 0 | 34 | 23 | 63 | 120.00 | 0.028 |
| cml_e3 | 0 | 0 | 0 | 16 | 7 | 6 | 29.00 | 0.038 |
| vansd | 7 | 28 | 20 | 0 | 1 | 0 | 0.02 | 0.043 |
| bl2b_tle | 0 | 0 | 0 | 17 | 6 | 7 | 30.00 | 0.047 |
| vanxyc | 8 | 20 | 7 | 0 | 0 | 0 | 0.03 | 0.049 |
| srmb | 69 | 54 | 58 | 434 | 410 | 1054 | 10.43 | 0.053 |
| ant2ib | 0 | 1 | 0 | 165 | 35 | 107 | 153.50 | 0.053 |
| ant2ia | 0 | 0 | 0 | 18 | 3 | 18 | 39.00 | 0.060 |
| ykkd | 0 | 0 | 0 | 23 | 4 | 27 | 54.00 | 0.064 |
| smee | 0 | 1 | 0 | 113 | 16 | 107 | 118.00 | 0.067 |
| ermx | 0 | 0 | 1 | 14 | 10 | 37 | 30.50 | 0.076 |
| aac3iia | 16 | 6 | 4 | 0 | 0 | 0 | 0.04 | 0.080 |
| tetm | 0 | 0 | 0 | 4 | 1 | 7 | 12.00 | 0.082 |
| oleb | 244 | 469 | 347 | 577 | 443 | 655 | 1.58 | 0.084 |
| ant4iia | 0 | 0 | 0 | 96 | 12 | 127 | 235.00 | 0.085 |
| tetv | 0 | 0 | 0 | 38 | 12 | 10 | 60.00 | 0.091 |
| bcra | 0 | 0 | 0 | 8 | 5 | 23 | 36.00 | 0.097 |
| bl2c_bro | 0 | 0 | 0 | 16 | 3 | 29 | 48.00 | 0.100 |
| ceob | 0 | 0 | 0 | 3 | 3 | 11 | 17.00 | 0.101 |
| ereb | 0 | 0 | 0 | 1 | 22 | 15 | 38.00 | 0.109 |
| ermf | 0 | 0 | 0 | 1 | 1 | 0 | 2.00 | 0.116 |
| vgbb | 0 | 0 | 0 | 6 | 0 | 7 | 13.00 | 0.118 |
| ermg | 0 | 0 | 0 | 26 | 0 | 35 | 61.00 | 0.125 |
| vanyb | 0 | 3 | 2 | 0 | 0 | 0 | 0.20 | 0.132 |
| tetz | 0 | 0 | 0 | 2 | 0 | 3 | 5.00 | 0.132 |
| erma | 0 | 0 | 1 | 4 | 1 | 9 | 7.00 | 0.140 |
| tet | 1 | 0 | 3 | 20 | 280 | 581 | 176.20 | 0.146 |
| bl2d_lcr1 | 0 | 0 | 0 | 1 | 2 | 7 | 10.00 | 0.147 |
| dfra20 | 0 | 0 | 0 | 0 | 6 | 11 | 17.00 | 0.149 |
| vgaa | 1 | 1 | 2 | 5 | 19 | 5 | 5.80 | 0.149 |
| aph6ia | 0 | 0 | 0 | 16 | 21 | 94 | 131.00 | 0.158 |
| vand | 0 | 4 | 2 | 0 | 0 | 0 | 0.17 | 0.158 |
| dfra26 | 0 | 3 | 3 | 16 | 130 | 319 | 66.43 | 0.158 |
| ykkc | 0 | 0 | 0 | 68 | 6 | 21 | 95.00 | 0.165 |
| tetq | 0 | 0 | 0 | 25 | 6 | 3 | 34.00 | 0.175 |
| mexw | 0 | 0 | 0 | 34 | 24 | 182 | 240.00 | 0.192 |
| tet34 | 0 | 0 | 1 | 3 | 8 | 35 | 23.00 | 0.206 |
| lnub | 0 | 0 | 0 | 1 | 6 | 28 | 35.00 | 0.232 |
| tet36 | 0 | 0 | 0 | 0 | 19 | 78 | 97.00 | 0.241 |
| bl1_pse | 1 | 0 | 3 | 1 | 5 | 13 | 3.80 | 0.241 |
| otra | 18 | 93 | 49 | 36 | 360 | 160 | 3.45 | 0.245 |
| ermw | 0 | 0 | 0 | 4 | 4 | 42 | 50.00 | 0.259 |
| mexd | 1 | 0 | 0 | 2 | 3 | 23 | 14.00 | 0.259 |
| ant3ia | 0 | 0 | 1 | 2 | 0 | 6 | 4.00 | 0.263 |
| lmrb | 0 | 0 | 0 | 1 | 11 | 69 | 81.00 | 0.272 |
| msra | 0 | 0 | 0 | 0 | 4 | 24 | 28.00 | 0.277 |
| mdte | 0 | 0 | 0 | 1 | 6 | 0 | 7.00 | 0.277 |
| mexa | 0 | 0 | 0 | 0 | 4 | 28 | 32.00 | 0.289 |
| smeb | 0 | 0 | 0 | 7 | 0 | 1 | 8.00 | 0.289 |
| adec | 0 | 0 | 1 | 0 | 18 | 127 | 72.50 | 0.293 |
| bl2be_per | 0 | 0 | 0 | 1 | 0 | 8 | 9.00 | 0.299 |
| tett | 0 | 0 | 1 | 11 | 34 | 377 | 211.00 | 0.301 |
| vgba | 0 | 0 | 0 | 0 | 1 | 11 | 12.00 | 0.318 |
| mexe | 0 | 0 | 1 | 4 | 123 | 6 | 66.50 | 0.326 |
| bl2d_r39 | 0 | 0 | 0 | 5 | 4 | 134 | 143.00 | 0.331 |
| vatd | 3 | 2 | 2 | 21 | 1 | 5 | 3.38 | 0.337 |
| vgab | 48 | 2 | 13 | 1 | 58 | 171 | 3.59 | 0.343 |
| vanre | 12 | 24 | 11 | 19 | 17 | 26 | 1.29 | 0.373 |
| adeb | 0 | 0 | 0 | 0 | 0 | 86 | 86.00 | 0.374 |
| aph6ic | 0 | 0 | 0 | 0 | 79 | 0 | 79.00 | 0.374 |
| dfra13 | 0 | 0 | 0 | 0 | 0 | 24 | 24.00 | 0.374 |
| qac | 0 | 0 | 0 | 0 | 0 | 15 | 15.00 | 0.374 |
| mphb | 0 | 0 | 0 | 7 | 0 | 0 | 7.00 | 0.374 |
| fosa | 0 | 0 | 0 | 0 | 0 | 3 | 3.00 | 0.374 |
| tet30 | 0 | 0 | 0 | 3 | 0 | 0 | 3.00 | 0.374 |
| aph6ib | 0 | 0 | 0 | 0 | 1 | 0 | 1.00 | 0.374 |
| bl2a_nps | 0 | 0 | 0 | 0 | 1 | 0 | 1.00 | 0.374 |
| tete | 0 | 0 | 0 | 0 | 0 | 1 | 1.00 | 0.374 |
| arna | 0 | 1 | 2 | 0 | 0 | 1 | 0.25 | 0.374 |
| vansg | 0 | 38 | 2 | 3 | 2 | 0 | 0.12 | 0.399 |
| vata | 129 | 40 | 48 | 75 | 32 | 44 | 0.69 | 0.519 |
| vanrc | 3 | 0 | 0 | 0 | 8 | 0 | 2.00 | 0.590 |
| vate | 83 | 113 | 89 | 196 | 57 | 93 | 1.21 | 0.658 |
| tetx | 0 | 0 | 1 | 0 | 0 | 2 | 1.00 | 0.678 |
| vanse | 5 | 2 | 4 | 1 | 0 | 7 | 0.67 | 0.693 |
| vane | 2 | 5 | 0 | 4 | 2 | 0 | 0.75 | 0.866 |
| vatc | 36 | 10 | 16 | 32 | 26 | 1 | 0.94 | 0.939 |
| catb5 | 8 | 1 | 8 | 2 | 1 | 14 | 0.94 | 1.000 |

**Addation: Fold change＞1 means up-regulation, Fold change＜1 means down-regulation.**
