# Supplementary material for: Shifts in microbial community, pathogenicity‐related genes and antibiotic resistance genes during dairy manure piled up
Source: Microb Biotechnol. 2020 Mar 23;13(4):1039–53. doi: 10.1111/1751-7915.13551 (PMC7264890; doi:10.1111/1751-7915.13551)
Supplement: Supplementary file 10 — Table S6. Differential number of contigs (FPKM) in multiple antibiotic resistances between group F and group M. [file MBT2-13-1039-s010.docx]

**Table S5. Differential number of contigs(FPKM) in multiple** **antibiotic resistance between Group F and Group M.**

| **Antibiotic** | **Number of** **contigs(FPKM)** | | | | | | **Fold Change** | **P-value** |
| --- | --- | --- | --- | --- | --- | --- | --- | --- |
|  | **F1** | **F2** | **F3** | **M1** | **M2** | **M3** |  |  |
| aminoglycoside \|\| glycylcycline | 20 | 14 | 23 | 0 | 0 | 2 | 0.03 | 0.003 |
| 6_n_netilmicin \|\| dibekacin \|\| gentamicin \|\| netilmicin \|\| tobramycin | 0 | 0 | 0 | 9 | 8 | 14 | 31 | 0.005 |
| lincosamide \|\| macrolide \|\| streptogramin_b | 483 | 838 | 597 | 2418 | 2179 | 4288 | 4.63 | 0.026 |
| teicoplanin \|\| vancomycin | 11 | 34 | 25 | 0 | 1 | 0 | 0.01 | 0.026 |
| amikacin \|\| dibekacin \|\| isepamicin \|\| netilmicin \|\| sisomicin \|\| tobramycin | 0 | 0 | 0 | 10 | 17 | 6 | 33 | 0.027 |
| cephalosproin \|\| penicillin | 0 | 0 | 0 | 17 | 6 | 7 | 30 | 0.047 |
| dibekacin \|\| gentamicin \|\| kanamycin \|\| sisomicin \|\| tobramycin | 0 | 1 | 0 | 183 | 38 | 125 | 173 | 0.052 |
| dibekacin \|\| gentamicin \|\| netilmicin \|\| sisomicin \|\| tobramycin | 16 | 6 | 4 | 34 | 23 | 63 | 4.44 | 0.066 |
| amikacin \|\| isepamicin \|\| tobramycin | 0 | 0 | 0 | 96 | 12 | 127 | 235 | 0.085 |
| carbenicillin \|\| penicillin | 0 | 0 | 0 | 16 | 3 | 29 | 48 | 0.100 |
| erythromycin \|\| fluoroquinolone \|\| glycylcycline \|\| roxithromycin | 1 | 0 | 0 | 2 | 3 | 23 | 14 | 0.259 |
| spectinomycin \|\| streptomycin | 0 | 0 | 1 | 2 | 0 | 6 | 4 | 0.263 |
| doxorubicin \|\| erythromycin | 0 | 0 | 0 | 1 | 6 | 0 | 7 | 0.277 |
| aminoglycoside \|\| beta_lactam \|\| fluoroquinolone \|\| tetracycline \|\| tigecycline | 0 | 0 | 0 | 0 | 4 | 28 | 32 | 0.289 |
| e_cephalosproin \|\| monobactam \|\| n_cephalosproin \|\| penicillin | 0 | 0 | 0 | 1 | 0 | 8 | 9 | 0.299 |
| cloxacillin \|\| penicillin | 0 | 0 | 0 | 6 | 6 | 141 | 153 | 0.320 |
| aminoglycoside \|\| chloramphenicol | 0 | 0 | 1 | 0 | 18 | 213 | 115.5 | 0.324 |
| chloramphenicol \|\| fluoroquinolone | 0 | 0 | 1 | 4 | 123 | 6 | 66.5 | 0.326 |
| aminoglycoside \|\| glycylcycline | 20 | 14 | 23 | 0 | 0 | 2 | 0.03 | 0.003 |
